# A genome-wide positioning systems network algorithm for in silico drug repurposing

**DOI:** 10.1038/s41467-019-10744-6

**Published:** 2019-08-02

**Authors:** Feixiong Cheng, Weiqiang Lu, Chuang Liu, Jiansong Fang, Yuan Hou, Diane E. Handy, Ruisheng Wang, Yuzheng Zhao, Yi Yang, Jin Huang, David E. Hill, Marc Vidal, Charis Eng, Joseph Loscalzo

**Affiliations:** 1 Genomic Medicine Institute, Lerner Research Institute, Cleveland Clinic, Cleveland, OH 44195 USA; 2 Department of Molecular Medicine, Cleveland Clinic Lerner College of Medicine, Case Western Reserve University, Cleveland, OH 44195 USA; 3 Case Comprehensive Cancer Center, Case Western Reserve University School of Medicine, Cleveland, OH 44106 USA; 4 Shanghai Key Laboratory of Regulatory Biology, Institute of Biomedical Sciences and School of Life Sciences, East China Normal University, Shanghai, 200241 China; 5 Alibaba Research Center for Complexity Sciences, Hangzhou Normal University, 311121 Hangzhou, China; 6 Department of Medicine, Brigham and Women’s Hospital, Harvard Medical School, Boston, MA 02115 USA; 7 Shanghai Key Laboratory of New Drug Design, School of Pharmacy, East China University of Science and Technology, 200237 Shanghai, China; 8 Synthetic Biology and Biotechnology Laboratory, State Key Laboratory of Bioreactor Engineering, Shanghai Collaborative Innovation Center for Biomanufacturing Technology, 200237 Shanghai, China; 9 Center for Cancer Systems Biology (CCSB), Dana-Farber Cancer Institute, Boston, MA 02215 USA; 10 Department of Genetics, Blavatnik Institute, Harvard Medical School, 77 Avenue Louis Pasteur, Boston, MA 02115 USA; 11 Taussig Cancer Institute, Cleveland Clinic, Cleveland, OH 44195 USA; 12 Department of Genetics and Genome Sciences, Case Western Reserve University School of Medicine, Cleveland, OH 44106 USA

**Keywords:** Drug discovery, Genetics, Systems biology

## Abstract

Recent advances in DNA/RNA sequencing have made it possible to identify new targets rapidly and to repurpose approved drugs for treating heterogeneous diseases by the ‘precise’ targeting of individualized disease modules. In this study, we develop a Genome-wide Positioning Systems network (GPSnet) algorithm for drug repurposing by specifically targeting disease modules derived from individual patient’s DNA and RNA sequencing profiles mapped to the human protein-protein interactome network. We investigate whole-exome sequencing and transcriptome profiles from ~5,000 patients across 15 cancer types from The Cancer Genome Atlas. We show that GPSnet-predicted disease modules can predict drug responses and prioritize new indications for 140 approved drugs. Importantly, we experimentally validate that an approved cardiac arrhythmia and heart failure drug, ouabain, shows potential antitumor activities in lung adenocarcinoma by uniquely targeting a HIF1α/LEO1-mediated cell metabolism pathway. In summary, GPSnet offers a network-based, in silico drug repurposing framework for more efficacious therapeutic selections.

## Abstract (web summary)

Identification of disease modules in the human interactome can guide more efficacious therapeutic selections. Here, the authors introduce a network-based methodology to identify individualized disease modules by mapping patients’ DNA and RNA sequencing profiles to the interactome, enabling prediction of cancer type-specific drug responses.

## Introduction

After the completion of the human genome project in 2003, there has been unexpected enthusiasm for how genetics and genomics would inform drug discovery and development^1^. Although it is in its infancy, the use of genomics in the drug discovery and development pipeline has generated some successes^2,3^. For example, proprotein convertase subtilisin/kexin type 9 (PCSK9), first discovered by human genetics studies in 2003, has generated great interest in genomics-informed drug discovery in cardiovascular disease^4^. However, the overall clinical efficacy of genome-derived approved drugs has remained limited owing to the heterogeneity of complex diseases.

Drug development in the genomics era has become a highly integrated systems pipeline in which complementary multi-omics and computational methods are used^3^. Recent technological and computational advances in genomics and systems biology have now made it possible to identify new druggable targets and therapeutic agents by uniquely targeting cancer type-specific mechanisms (e.g., perturbed pathways in the disease module) that cause or contribute to human disease^5–8^. For example, network-based approaches have offered possibilities for drug repurposing^8^, target identification^9^, and combination therapy^10^ by quantifying the proximity of drug targets and disease proteins in the human protein–protein interactome. It remains unclear as to how generalizable these network-based disease module identification methodologies are in exploiting the wealth of massive multi-omics data from genomics studies and offering novel insights into cancer type-specific mechanisms. Were they to be successful, we would ultimately be able to target precisely human disease pathways, promoting the development of precision medicine.

In this study, we present a novel network-based disease module identification and in silico drug repurposing methodology, denoted the Genome-wide Positioning Systems network (GPSnet) algorithm. Specifically, we demonstrate the feasibility of individualized disease module identification by integrating large-scale DNA sequencing and transcriptome (RNA-seq) profiling across approximately 5000 human tumor genomes to the human protein–protein interactome. We find that gene expression of disease modules identified by GPSnet can predict drug responses in cancer cell lines with high accuracy. Importantly, we show that GPSnet can be used for in silico drug repurposing by uniquely targeting the specific disease module (i.e., cancer type-specific module) through combining network proximity measure and gene-set enrichment analysis (GSEA) approaches. Furthermore and importantly, we validate these network-based predictions experimentally. From a translational perspective, if broadly applied, GPSnet offers a powerful network-based tool for target identification and drug repurposing. We believe this approach can minimize the translational gap between genomics studies and drug development, currently a significant bottleneck in precision medicine.

## Results

### Modularity of highly mutated genes in the human interactome

**Fig. 1:**
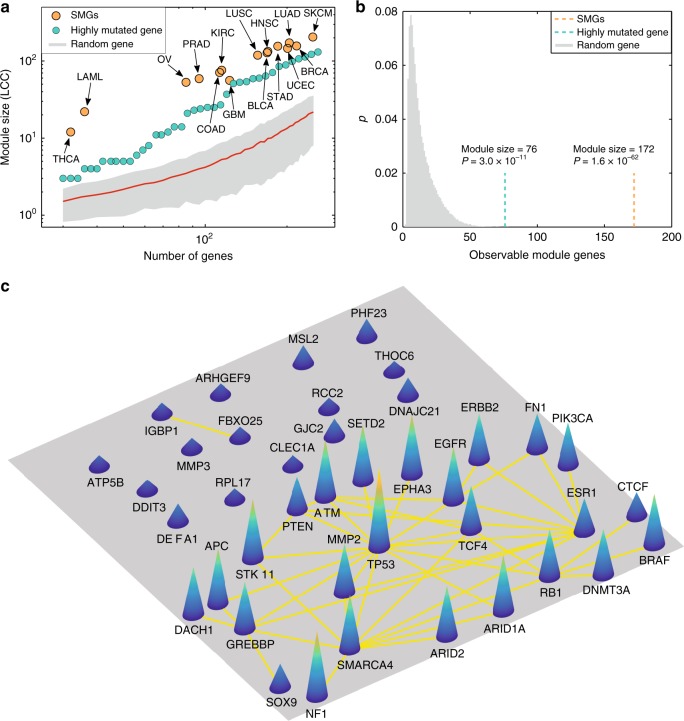
Proof-of-concept of cancer type-specific disease module. **a** Both significantly mutated genes (SMGs, Supplementary Data 1) identified by statistical approaches and highly mutated genes ranked by mutation frequency form a significant largest connected component (LCC) compared to random genes with matching connectivity (degree) distributed in the human protein–protein interactome, across 15 cancer types (Supplementary Fig. 6). **b** The sizes of the LCC of SMGs and of highly mutated proteins are shown for lung adenocarcinoma (LUAD). The *y*-axis (*p*) denotes the fraction of the number of random modules against the total number of permutations. The observed module sizes, 172 (SMGs, orange line) and 76 (highly mutated gene, cyan line), are significantly larger than the random expectation. The *P* value was calculated by permutation test. **c** A subgraph illustrating a subnetwork of highly mutated genes versus genes with low mutation frequency in LUAD. The full names of the 15 cancer types are provided in the main text

Disease proteins are not scattered randomly in the human protein–protein interactome, but form one or several connected subgraphs, defining the disease module^7^. Yet, whether the mutant proteins directly derived from individual patient sequencing data (e.g., whole-exome sequencing) form a statistically significant module in the human protein–protein interactome (or subnetwork in a disease module) remains unknown. To test this hypothesis, we first collected the significantly mutated genes (SMGs) identified from large-scale genome sequencing projects across 15 cancer types (Supplementary Data 1, see Methods section). We found that SMGs form highly connected subgraphs in the human protein–protein interactome across all 15 cancer types (Fig. 1a). In lung adenocarcinoma (LUAD), 83.1% of genes (172/207, *P* = 1.6 × 10^−62^ [permutation test]) form the largest connected component that is statistically signifcantly clustered compared to the same number of randomly selected genes with similar connectivity (degree) as the original SMGs in the human interactome (Fig. 1b). Interestingly, we found that genes with high somatic mutation frequency also tend to form highly connected subgraphs compared to the same fraction of random gene sets with the same degree distribution as the highly mutated genes in the human interactome. For example, mutated genes with high somatic mutation frequency form a significant module (*P* = 3.0 × 10^−^^11^, Fig. 1b) in LUAD, although the module size of highly mutated genes is less than that of SMGs. Previous studies have suggested that well-known cancer genes/proteins often have high connectivity in the literature-derived human protein–protein interactome. To inspect potential literature data bias, we performed the same network modularity analysis using the high-throughput systematic interactome (see Methods section) identified by the (unbiased) yeast two-hybrid (Y2H) screening assays previously published^11^. We found that the SMGs and highly mutated genes form a significant module in this more limited but unbiased interactome, as well (Supplementary Note 1 and Supplementary Fig. 1), suggesting low literature data bias.

Figure 1c illustrates the connectivity of products of several highly mutated genes (such as *EGFR*, *TP53*, and *NF1*) compared to genes with low somatic mutation frequency in LUAD. These observations are consistent with previous network-based studies showing that experimentally reported cancer proteins are more likely to connect to each other in the human interactome than to noncancer proteins^12–14^. The strong modularity of gene products with high mutation frequency led us to develop new network-based methodologies to identify cancer type-specific disease modules by mapping individual patient’s DNA sequencing data to the human protein–protein interactome disease network model.

### Pipeline of GPSnet

**Fig. 2:**
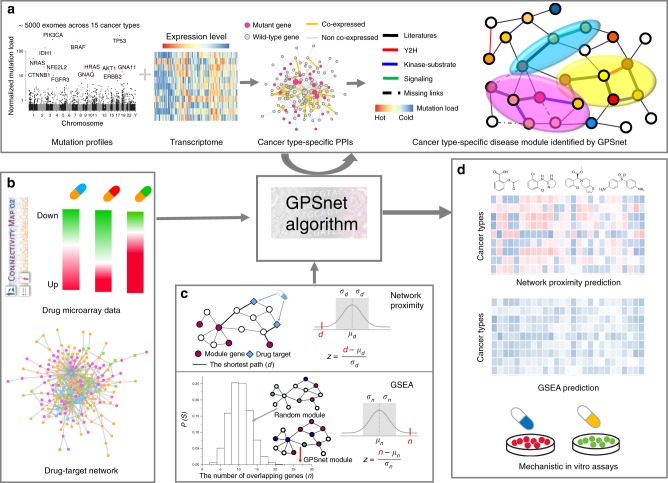
A diagram illustrating the pipeline of GPSnet for in silico drug repurposing. The GPSnet pipeline contains three key steps: **a** network-based identification of cancer type-specific disease modules by integrating patient’s DNA sequencing (somatic mutations) and transcriptome (RNA-seq) data into the human protein–protein interactome. We collected the human protein–protein interactions (PPIs) by assembling multiple types of experimental data, and built the cancer type-specific (co-expression) PPI network based on tumors’ RNA-seq data (see Methods section). The Manhattan plot shows the pan-cancer mutation load distribution. The heatmap shows gene expression (RNA-seq) across tumor samples; red denotes high expression levels and blue denotes low-expression levels. **b**, **c** Performing both a network proximity analysis (quantifying the network distance of drug targets to cancer type-specific disease modules from **a** in the human protein–protein interactome) and gene-set enrichment analysis (GSEA) by searching drug-up/downregulated genes from drug-induced transcriptome data (Connectivity Map) in human cell lines in cancer type-specific disease modules; and **d** in silico drug repurposing and experimental validation

Here, we present GPSnet, an integrated, network-based methodology for cancer type-specific disease module identification and in silico drug repurposing. Figure 2 illustrates the pipeline of the GPSnet algorithm, which contains two main components: (a) cancer type-specific disease module identification by integrating whole-exome sequencing (somatic mutation) and transcriptome (RNA-seq) profiling into the human protein–protein interactome (Fig. 2a), and (b) in silico drug repurposing that targets uniquely cancer type-specific disease modules generated from the first step by implementing both GSEA and network proximity approaches (Fig. 2b, c) complemented by mechanistic in vitro validation (Fig. 2d) in human cell lines (see Methods section).

Figure 1 shows that genes with high mutation frequency form highly connected subgraphs called disease modules. In addition, we find that genes in the subnetwork identified from the RNA-seq database co-expressed protein–protein interactome are highly mutated, and SMGs are significantly enriched in the subnetworks identified from RNA-seq data, as well (Supplementary Note 2 and Supplementary Figs. 2, 3). Here, we thereby define a module as a subgraph within the human protein–protein interactome (see Methods section) and calculate the score of the module *M* as

1 $$Z_m = \frac{{\mathop {\sum }\nolimits_{i \in M} \,(s(i) - \mu )}}{{\sqrt m }}{,}$$

where *m* is the number of genes in module *M*, *s*(*i*) is the network-predicted mutation frequency of gene *i* normalized by a gene’s cDNA sequence length, and *μ* is the average mutation frequency over the whole gene set for a specific cancer type after network smoothing using the random walk with restart algorithm (see Supplementary Note 3). We denote Γ_*M*_ as the set of genes that interact with module *M* in the human protein–protein interactome. Specifically, we first randomly select a protein (gene) as a “seed protein.” For each gene $i \in {\mathrm{\Gamma }}_M$, we next calculate its connectivity significance^15^ in the human protein–protein interactome as

2 $$P(i) = \mathop {\sum }\limits_{k = k_m}^{k_i} \,\frac{{\left( {\begin{array}{*{20}{c}} m \\ k \end{array}} \right)\left( {\begin{array}{*{20}{c}} {N - m} \\ {k_i - k} \end{array}} \right)}}{{\left( {\begin{array}{*{20}{c}} N \\ {k_i} \end{array}} \right)}}{,}$$

where $k_i$ is the degree of gene *i*, *m* is the number of genes in the module, $k_m$ is the number of module genes that link to gene *i*, and *N* is the total number of genes in the gene set. For each seed $i \in \Gamma _M$, we further calculate the expanded module score if gene *i* is added to the module as

3 $$Z_{m + 1}(i) = \frac{{\left( {s(i) - \mu } \right) + \mathop {\sum }\nolimits_{i \in M} \,(s(i) - \mu )}}{{\sqrt {m + 1} }}{.}$$

The candidate gene *i* will be added to the module to build a new module by satisfying three criteria: (a) *P*(*i*) in Eq. (2) is less than 0.05 (connectivity significance), (b) this candidate gene is significantly co-expressed (*P* value < 0.05, *F*-statistic) within the neighborhood of the raw module based on RNA-seq profiles of tumor samples (see Methods section), and (c) $Z_{m + 1}(i) > Z_m$. We repeat steps (a) and (c) until no more genes can be added by these criteria. In this study, we built ~60,000 raw modules for each cancer type by randomly selecting each seed gene in the human interactome approximately five times. Finally, we assembled the top 1% (approximately 300) modules with the highest module scores and defined the largest connected component^7^ by removing the isolated genes (less than 5% of isolated genes [nodes] across all 15 cancer types) to define the final disease module for each cancer type (Supplementary Fig. 4). Details are provided in the Supplementary Note 3.

### Cancer type-specific disease module identified by GPSnet

**Fig. 3:**
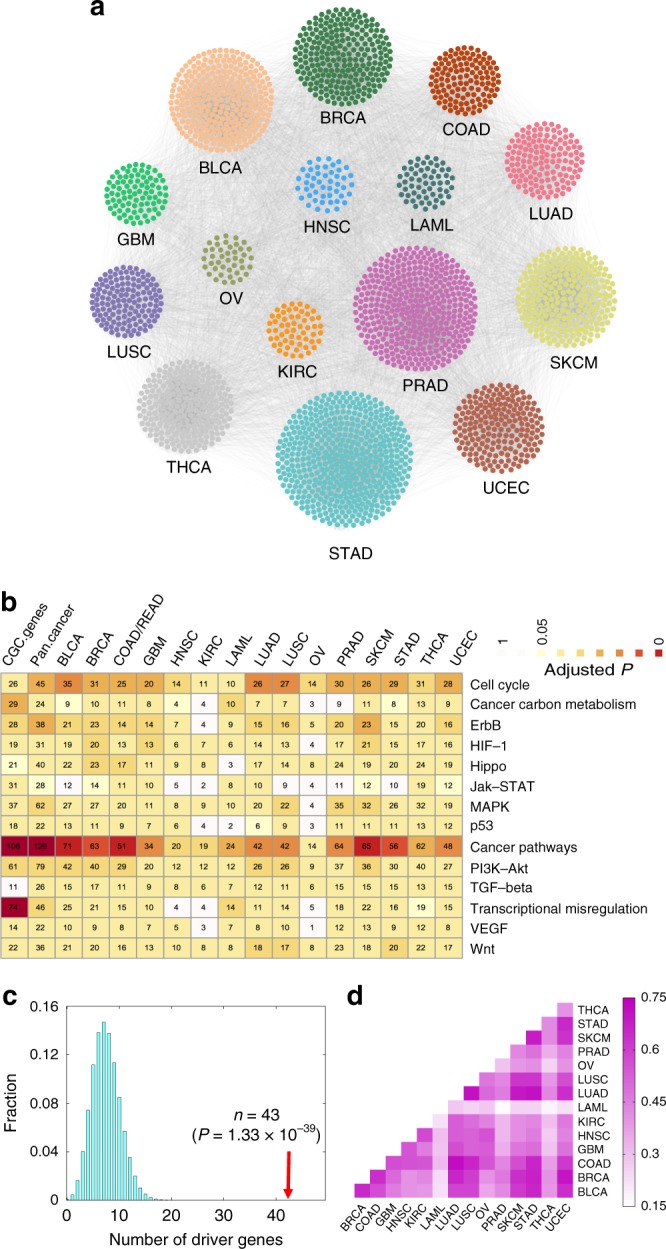
Network-based identification and validation of cancer type-specific disease modules via GPSnet. **a** Pan-cancer analysis of cancer type-specific disease modules (Supplementary Data 2) identified by GPSnet across 15 cancer types. **b** Canonical cancer pathway enrichment analysis for the cancer type-specific disease modules identified by GPSnet across 15 cancer types. **c** Known cancer driver genes (named significantly mutated genes, Supplementary Data 1) are appreciably enriched in cancer type-specific disease modules in breast cancer. The cancer enrichment analyses for other cancer types are provided in Supplementary Fig. 6. The *P* value was computed by the permutation (randomization) test (see Methods section). **d** A heat map illustrating modularity similarity between known cancer driver genes (named significantly mutated genes) and the module genes identified by GPSnet across 15 cancer types. Modularity similarity (color key) is measured by the Jaccard-index $J = \left| {A \cap B} \right|/\left| {A \cup B} \right|$, where gene set A represent the known cancer driver genes (*Y*-axis) and gene set B represent module genes identified by GPSnet (*X*-axis). The full names of the 15 cancer types are provided in main text

Via GPSnet, we identify computationally the final cancer type-specific disease modules for all 15 cancer types by integrating iteratively the patients’ whole-exome sequencing and RNA-seq data into the human protein–protein interactome (Fig. 2a). Figure 3a shows the network visualization of disease modules identified by GPSnet across 15 cancer types: urothelial bladder carcinoma (BLCA), invasive breast carcinoma (BRCA), colorectal adenocarcinoma (COAD), glioblastoma multiforme (GBM), head and neck squamous cell carcinoma (HNSC), kidney renal clear cell carcinoma (KIRC), acute myeloid leukemia (LAML), lung adenocarcinoma (LUAD), lung squamous cell carcinoma (LUSC), ovarian serous cystadenocarcinoma (OV), prostate adenocarcinoma (PRAD), skin cutaneous melanoma (SKCM), stomach adenocarcinoma (STAD), papillary thyroid carcinoma (THCA), and uterine corpus endometrial carcinoma (UCEC). The distributions of overlapping genes across different cancer types are provided in Supplementary Fig. 5. We find that most cancer types share multiple genes with other cancer types; however, several cancer types, including prostate adenocarcinoma (PRAD, 84 unique genes), papillary thyroid carcinoma (THCA, 108 unique genes), and urothelial bladder carcinoma (BLCA, 50 unique genes), have high numbers of unique module genes compared to other cancers, suggesting unique biological pathways for these cancer types. The detailed data for 15 cancer type-specific disease modules identified by GPSnet are provided in Supplementary Data 2.

We next attempted to validate the GPSnet-identified disease modules via two approaches: (a) canonical disease pathway enrichment analysis, and (b) well-known disease-associated gene enrichment analysis. As shown in Fig. 3b, canonical cancer pathway enrichment analyses showed that genes in the GPSnet-predicted disease modules are significantly enriched in various canonical cancer pathways, which is comparable to the experimentally validated cancer genes from the Cancer Gene Census (CGC) database^16^. We further find that the known SMGs (Supplementary Data 1) are significantly enriched in the disease modules identified by GPSnet across all 15 cancer types (Fig. 3c, and Supplementary Fig. 6). For BRCA, SMGs are enriched in the identified breast cancer modules, which is significantly stronger than a random gene set with the same connectivity (degree) distribution (*P* = 1.33 × 10^−39^, Fig. 3c). We calculate the module similarity (Jaccard-Index) across 15 cancer types in Fig. 3d. We find that the disease modules of several cancer types are very similar (such as LUAD and LUSC), while some cancer types have unique disease modules, such as LAML and PRAD, indicating both common mechanisms and unique pathways identified by GPSnet. We further collect the disease-associated genes from several publicly available databases (Supplementary Data 3, see Methods section). Supplementary Fig. 7 shows that cancer-associated genes are significantly enriched in the cancer type-specific disease modules identified by GPSnet, as well. To assess the impact of the literature data bias of the human protein–protein interactome, we re-identify cancer type-specific disease modules for 15 cancer types using the unbiased, Y2H high-throughput systematic human interactome^11^. We find that known SMGs are significantly enriched in the cancer type-specific disease modules identified from the unbiased, systematic interactome by GPSnet, as well, revealing the low literature data bias for GPSnet (Supplementary Fig. 8). Altogether, cancer type-specific disease modules are highly enriched in the known cancer genes and canonical cancer pathways, suggesting potential pathobiological implications of GPSnet-based analysis.

### Identification of new pharmacogenomics biomarkers by GPSnet

To examine further the potential pharmacogenomics application of GPSnet, we downloaded robust multi-array (RMA) gene expression profiles and drug response data (IC_50_, the half maximal inhibitory concentration) across 1065 cell lines from the Genomics of Drug Sensitivity in Cancer (GDSC) database^17^. We then built regression models to predict drug responses (IC_50_) based on RMA gene expression profiles of GPSnet-identified disease modules (see Methods section) for three specific cancer types: BRCA, LUAD, and SKCM. We focused on seven drugs across those three cancer types using subject matter expertise based on a combination of factors: (a) the highest variances of IC_50_ of each drug among over 1065 cell lines; (b) drugs approved by targeting specific pathways; and (c) cancer type with the highest number of tumor samples from TCGA. These seven selected drugs include enzastaurin, linifanib, olaparib, pictilisib, refametinib, selumetinib, and vemurafenib.

**Fig. 4:**
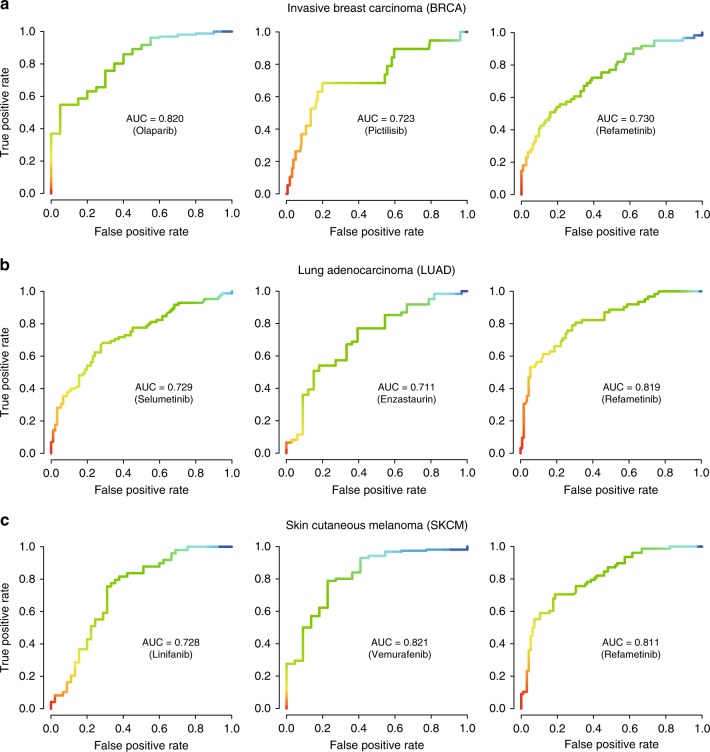
Pharmacogenomics validation for GPSnet-predicted disease modules. **a**–**c** The receiver operating characteristic (ROC) curves for seven selected drugs: enzastaurin, linifanib, olaparib, pictilisib, refametinib, selumetinib, and vemurafenib, for three specific cancer types, including invasive breast carcinoma (BRCA, **a**), lung adenocarcinoma (LUAD, **b**), and skin cutaneous melanoma (SKCM, **c**). Drug IC_50_ values were predicted based on regression models built by utilizing gene expression profiles of the GPSnet-identified cancer type-specific disease modules as feature vectors (see Methods section). The area under ROC curves (AUC) during 10-fold cross-validations are shown

We define cell lines whose IC_50_ value is higher than 10 µM as drug-resistant cell lines and the remainder as drug sensitive cell lines. As shown in Fig. 4, the area-under-the-receiver operating characteristic curves (AUC) ranges from 0.711 to 0.821 across 7 drugs with 10-fold cross-validation, revealing high performance in predicting drug responses by GPSnet-identified cancer type-specific disease modules. For example, olaparib, an inhibitor of poly (ADP-ribose) polymerase enzymes, is approved for treatment of breast cancer and ovarian cancer^18^. Figure 4a reveals that gene expression in the GPSnet-identified breast cancer disease module can predict olaparib’s response with AUC = 0.820. Vemurafenib, a B-Raf V600E targeted inhibitor, is approved for the treatment of late-stage melanoma^19^. We find that the GPSnet-predicted melanoma disease module can predict response to vemurafenib with an AUC = 0.821 (Fig. 4c). Refametinib, a MEK specific inhibitor, is currently in clinical trial for the treatment of RAS-mutant cancers^20^. Figure 4a–c shows that the GPSnet-predicted disease modules can accurately predict responses to refametinib in all three cancer types: BRCA (AUC = 0.730), LUAD (AUC = 0.819), and SKCM (AUC = 0.811). Altogether, gene expression in the GPSnet-disease modules offer potential pharmacogenomics biomarkers for assessment of drug resistance/sensitivity in multiple types of cancer.

### High druggability of disease modules identified by GPSnet

Previous studies have revealed that genes/proteins identified by genome sequencing studies, such as genome-wide association studies, often failed to identify novel druggable targets^21^. Integration of human PPIs, however, offers potential opportunities to identify druggable pathways by targeting adjacent mutant genes^21^. We further assessed whether genes in the GPSnet-predicted modules could offer more druggable targets compared to traditional statistics-based genome sequencing analysis. Interestingly, we find that gene products from the GPSnet-predicted modules are more likely to be targeted by approved drugs or clinically investigational drugs compared to SMGs that are identified by the statistics-based approaches alone (Supplementary Figs. 9, 10). For example, among 229 SMGs in breast cancer, only 17 are targeted by known drugs. However, among 236 genes in the GPSnet-predicted breast cancer module, 46 genes are targeted by known drugs, three-fold higher than SMGs (*P* < 0.0001, Fisher’s test). Furthermore, gene products in the GPSnet-identified modules are significantly targeted by known drugs compared to genome-wide drug targets’ distributions across all 15 cancer types (Supplementary Fig. 9). Importantly, the GPSnet-identified disease modules contain targets of drugs (Supplementary Data 4) known to treat this disease in two cancer types: LUAD (*P* < 0.0001, Fisher’s test, Supplementary Fig. 10) and BRCA (*P* < 0.0001, Fisher’s test, Supplementary Fig. 10). We further investigated drug target distribution in the human protein-protein interactome. As expected, we found that neighbors with significant connectivity to the known SMGs are more likely to be targeted by known drugs compared to SMGs only, or experimentally validated genes from the Cancer Gene Census database^16^ (Supplementary Fig. 11). Taken together, disease modules identified by GPSnet offer more druggable targets compared to traditional statistics-based genome sequencing analysis approaches. We, therefore, next turned to in silico drug repurposing by uniquely targeting GPSnet-predicted cancer type-specific disease modules derived from the human protein–protein interactome analysis.

### Uncovering new indications for approved drugs by GPSnet

**Fig. 5:**
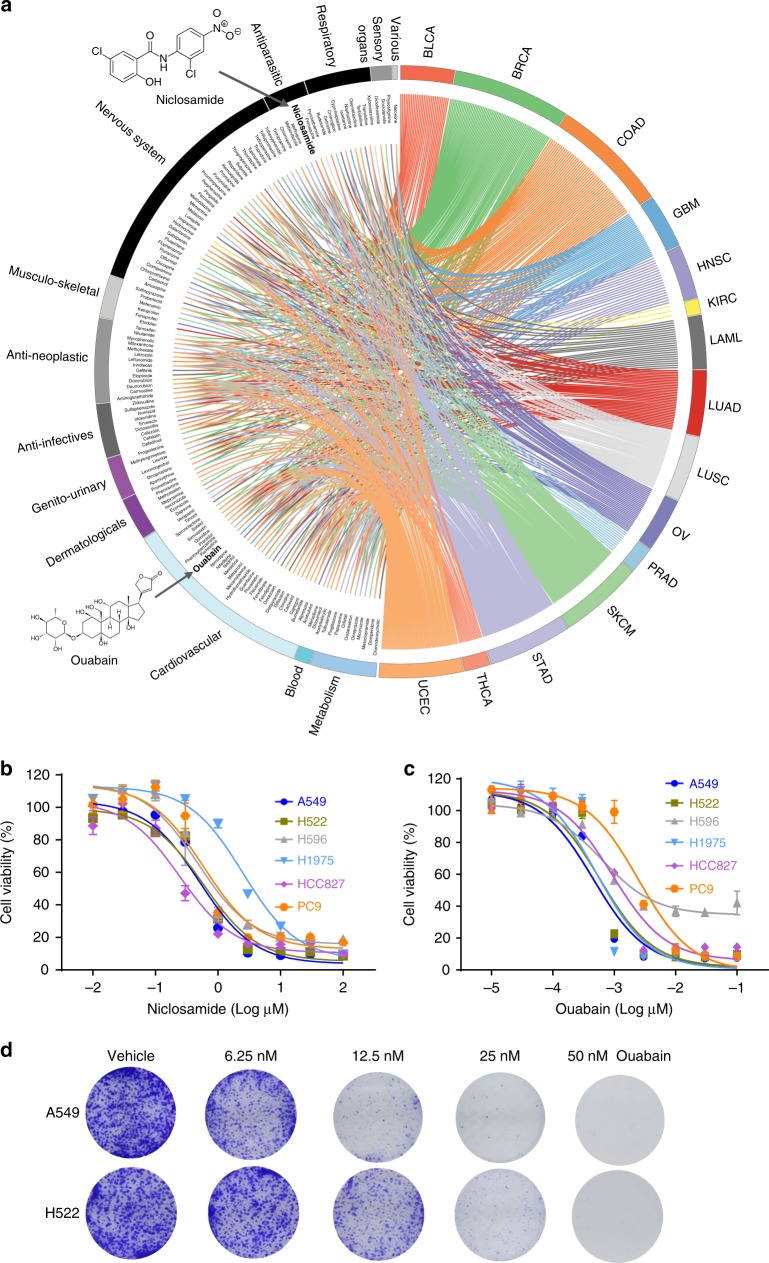
Network-based in silico drug repurposing and experimental validation. **a** A circos plot illustrating a global view of computationally predicted potential anticancer indications for 140 approved drugs across 15 cancer types, identified by both network proximity and gene-set enrichment analysis approaches. Drugs are grouped by their first-level Anatomical Therapeutic Chemical Classification (ATC) codes. Two overlapping drugs (ouabain and niclosamide) from the top 10 lists identified by both network proximity and GSEA approaches in lung adenocarcinoma (Supplementary Data 5) were selected for experimental validation. **b**, **c** Dose-response curves of ouabain (**b**) and niclosamide (**c**) in six representative non-small cell lung cancer (NSCLC) cell lines (A549, H522, H596, H1975, HCC827, and PC9). Cells were treated with a series of concentrations of ouabain and niclosamide for 48 h. The CellTiter 96 AQueous one solution cell proliferation kit was used to determine cell viability (see Methods section). Data are  represented as mean ± SEM (*n* = 3) and each experiment was performed at least three times in duplicate. **d** Effect of ouabain on the colony formation assay in two NSCLC cell lines (A549 and H522)

To identify novel indications for approved drugs, we utilize two complementary approaches: (i) a network proximity (*z*-score) measure that quantifies the relationship between cancer type-specific disease modules identified by GPSnet and drug targets in the human protein–protein interactome^8^ (see Methods section); and (ii) gene-set enrichment analysis (GSEA)^22^ that examines drug-induced gene signatures (up/downregulated genes in tumor cells before and after drug treatments) in the GPSnet-predicted disease module (Fig. 2c, see Methods section). Here, we investigate 1309 drugs in total that have known target information from publicly available databases or gene signatures from the Connectivity Map database^23^ (see Methods section). Figure 5a shows that 140 approved drugs (adjusted *P* < 0.01) are significantly associated with at least one cancer type identified by network proximity or GSEA approaches (Supplementary Data 5). For example, several known cancer drugs (such as gefitinib, etoposide, doxorubicin, and tamoxifen) defined by the first-level of the Anatomical Therapeutic Chemical Classification codes are correctly predicted by the network proximity approach compared to GSEA, revealing the power of the network approach for drug repurposing, consistent with our recent study^8^.

To examine further the performance of in silico drug repurposing in GPSnet, we inspected the top 10 predicted drugs by both network proximity and GSEA in LUAD. We find that two FDA-approved non-cancer drugs, niclosamide (an approved drug for the treatment of tapeworm infestations) and ouabain (an approved drug for treatment of cardiac arrhythmias and heart failure), are predicted to have significant associations (directionality of effect is not defined in this initial analysis) with LUAD by both network proximity and GSEA approaches (Fig. 5a and Supplementary Fig. 12). To test the antitumor effect of niclosamide and ouabain on non-small cell lung cancer (NSCLC), the MTS assay is performed to determine the cell proliferation capacity of 6 different cell lines: A549, H522, H596, H1975, HCC827, and PC9. We find that both niclosamide and ouabain show potential antitumor effects on these NSCLC cell lines (Fig. 5b, c). For example, ouabain is cytotoxic to all tested NSCLC cells in the nanomolar (nM) range: IC_50_ = 0.45 nM in A549 cells, IC_50_ = 0.58 nM in H522 cells, IC_50_ = 0.62 nM in H596 cells, IC_50_ = 0.52 nM in H1975 cells, IC_50_ = 0.94 nM in HCC827 cells, and IC_50_ = 2.48 nM in PC9 cells (Fig. 5c). Furthermore, a prolonged colony formation assay is performed to investigate whether ouabain causes irreversible growth arrest (Fig. 5d). We find that ouabain significantly impairs A549 and H522 cell growth, as demonstrated by a reduction in both colony number and colony size in the ouabain-treated group. Altogether, ouabain exhibits potential anti-proliferative efficacy and was, therefore, selected for further experimental evaluation.

### Ouabain inhibits HIF1α/LEO1 pathway in NSCLC cells

**Fig. 6:**
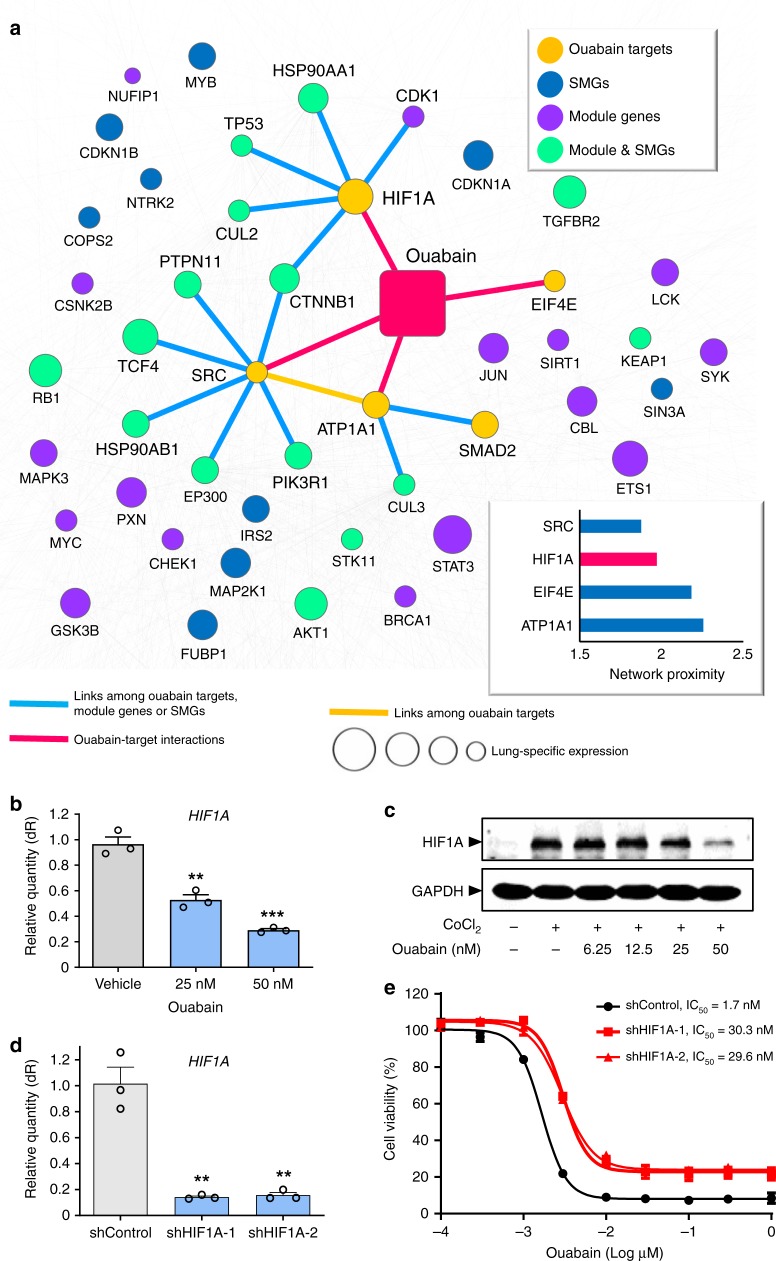
Network-based experimental validation of ouabain’s likely mechanism-of-action in non-small cell lung cancer (NSCLC). **a** A highlighted subnetwork reveals the inferred mechanism-of-action for ouabain’s anticancer effects on NSCLC by network analysis. The network distance (the shortest path) of four known ouabain targets to the cancer type-specific disease module of lung adenocarcinoma in the human protein–protein interactome is ranked for SRC, HIF1α, EIF4E, and ATP1A1. Yellow circles: known ouabain targets; blue circles: significantly mutated genes (SMGs) for lung adenocarcinoma (LUAD) based on data from The Cancer Genome Atlas (Supplementary Data 1); purple circles: LUAD-specific module genes identified by GPSnet; green circles: overlapping  genes between SMGs and LUAD-specific module genes. The size of circle nodes denote the gene expression level in lung compared to other 31 tissues based on RNA-seq data from GTEx database (see Methods section). The protein–protein interactions (PPIs) among ouabain targets, LUAD-specific module genes, and SMGs are labeled by blue links. The ouabain-target interactions are labeled by red links. Background light gray lines represent other edges in the dense PPIs unrelated to the LUAD-specific disease module. **b** Ouabain downregulates gene expression of *HIF1A* in a dose-dependent manner in A549 cells. **c** Western blot analysis of HIF1α protein expression (normalized by GAPHD) after CoCl_2_/ouabain treatment in A549 cells. The uncropped image for **c** is included as Supplementary Fig. 22. **d** shRNA significantly downregulates *HIF1A* gene expression in A549 cells. Differences between the two groups (**b** and **d**) were analyzed by Student’s *t*-test: *P-value < 0.05 and ***P-value < 0.001. **e** Cell viability reduction by ouabain is perturbed by two specific shRNA against *HIF1A*. Each experiment was performed at least three times in duplicate. All data is represented as mean ± SEM (*n* = 3) and each experiment was performed at least three times in duplicate

We next examined the mechanism-of-action of ouabain in NSCLC by network analysis on the lung-specific human protein–protein interactome. Currently, there are four reported targets (ATP1A1, EIF4E, HIF1α, and SRC) of ouabain in human cells. Figure 6a shows that SRC has the closest distance to the LUAD disease module identified by GPSnet, followed by HIF1α, EIF4E, and ATP1A1. Both SRC and EIF4E play essential roles in multiple cancers, including NSCLC^24,25^. We, therefore, examine whether HIF1α contributes to the antitumor effect of ouabain as it shows a closer network proximity to the LUAD module compared to the primary target, ATP1A1 (Fig. 6a). As predicted, ouabain downregulates both mRNA and protein expression of HIF1α in a dose-dependent manner in A549 cells (Fig. 6b, c). Knockdown of *HIF1A* by two distinct siRNAs reduces the response of ouabain in A549 cells from 5-fold to 6-fold (Supplementary Fig. 13). Moreover, knockdown of *HIF1A* by two distinct shRNAs reduces the response of ouabain in A549 cells approximately 17-fold (Fig. 6d, e). These observations reveal that HIF1α may contribute to the potential antitumor effect of ouabain in NSCLC cells.

**Fig. 7:**
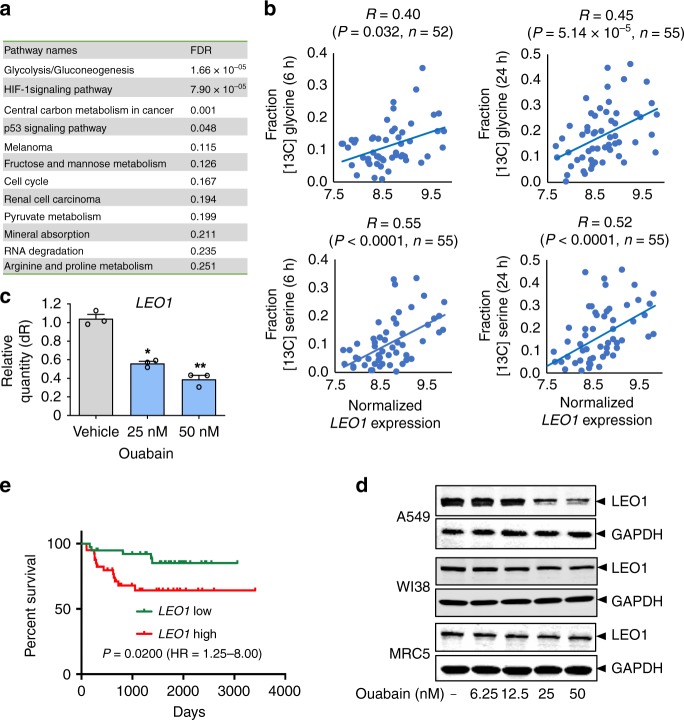
Ouabain regulates LEO1-mediated cell metabolism of non-small cell lung cancer (NSCLC) cells by bioinformatics and experimental validation. **a** Pathway enrichment analysis for the differentially expressed *HIF1A* target genes in lung adenocarcinoma patients. FDR: false discovery rate (also denoted the adjusted *p*-value for multiple testing using Bonferroni’s correction). **b** Correlation of *LEO1* expression with serine or glycine abundance in NSCLC cell lines (see Methods section and Supplementary Data 6). R: Pearson correlation coefficient. *P*-value (*P*) was calculated by *F*-statistics. *n* is the number of NSCLC cell lines were used for each correlation analysis. **c** Ouabain downregulates gene expression of *LEO1* in a dose-dependent manner in A549 cells. Each experiment was performed at least three times in duplicate. Differences between the two groups were analyzed by Student’s *t*-test: **P-value < 0.01 and ***P-value < 0.001. Data is represented as mean ± SEM (*n* = 3) and each experiment was performed at least three times in duplicate. **d** Western blot analysis of LEO1 protein expression (normalized by GAPDH) following ouabain treatment in A549 cells and two normal human lung fibroblasts cells (WI38 and MRC5). Uncropped images for **d** are included as Supplementary Fig. 23. **e** Survival analysis of *LEO1* gene expression in NSCLC patients (see Methods section). The *P*-value (*P*) was calculated by log-rank test. HR, hazard ratio

HIF1α activates the transcription of multiple genes involved in many human diseases, including cancer^26^. We next collect 719 putative HIF1α-targeted genes (Supplementary Data 6) identified by different high-throughput experiments or the curated literature (see Methods). We found that 251 HIF1α targeted genes (Supplementary Data 6) were differentially expressed in LUAD patients compared to normal control samples based on TCGA data^27^. KEGG pathway enrichment analysis shows that the top enriched pathways are cell metabolism-related pathways, including redox pathways, such as glycolysis/gluconeogenesis (adjusted *P* = 1.66 × 10^−5^) and central carbon metabolism, in cancer (adjusted *P* = 0.001, Fig. 7a). We further tested the effects of ouabain on redox metabolism using a highly responsive NAD+/NADH sensor, SoNar, reported in our previous study^28^. We found that ouabain at 10 or 20 nM reduced the NAD+/NADH ratio in A549 cells (Supplementary Fig. 14), suggesting that ouabain potentially targets cell (redox) metabolism in NSCLC cells.

We next collected genome-wide gene expression profiles from the CCLE database^29^ and the metabolite abundance on NSCLC cell lines from a recent study^30^. We computed the correlation of gene transcriptional activities and the serine/glycine abundance in approximately 70 NSCLC cell lines having both gene expression and metabolite abundance profiles. Among 251 HIF1α targeted genes with differential expression in LUAD patients, we found that the transcriptional activities of *LEO1* showed the highest correlation with [^13^C]serine or [^13^C]glycine abundance in NSCLC cell lines (Supplementary Data 6 and Fig. 7b). We tested the effect of ouabain on *LEO1* in A549 cells. Figure 7c, d shows that ouabain downregulates the expression of both mRNA and protein levels of LEO1 in A549 cells, whereas it has minor effect on LEO1 protein expression in two normal human lung fibroblast lines WI38 and MRC5. *LEO1*, encoding a component of RNA polymerase II-associated factor complex, has been reported to be involved in acute myelogenous leukemia^31^. Figure 7e shows that overexpression of *LEO1* is correlated with poor survival rate in LUAD patients (*P* = 0.02, Log-rank test, see Methods section). Taken together, these data show that regulation of HIF1α/LEO1-mediated cell metabolism may contribute to ouabain’s antitumor effects in NSCLC cells.

## Discussion

Molecularly targeted agents have demonstrated clinical efficacy and favorable toxicity profiles for the treatment of cancer compared to nonspecific cytotoxic chemotherapeutic agents. However, patients treated with those targeted agents often develop drug resistance due to clinically actionable genetic and epigenetic events in individuals, with survival typically prolonged by only a few months. Thus, there is clearly a need to identify new treatments with high efficacy and low toxicity profiles (e.g., by drug repurposing) for subsequent treatment of relapsed patients.

In this study, we developed an integrated interactome-based, systems pharmacology approach, GPSnet, for the systematic identification of novel targets (in cancer type-specific disease modules) or for repurposing approved drugs for development of new therapeutic strategies. To do so, we incorporated large-scale patient transcriptome profiles, whole-exome sequencing, drug-target network, and drug-induced microarray data into the human protein–protein interactome. We showed that both experimentally validated genes and canonical cancer pathways were significantly enriched in the disease modules identified by GPSnet. In addition, gene expression of GPSnet-identified disease modules can predict drug responses with reasonable accuracy (Fig. 4). We also showed that disease modules identified in the human protein–protein interactome are more likely to be druggable than the disease genes identified by traditional statistical approaches. Furthermore, we computationally identified multiple potential anticancer indications for 140 approved drugs across 15 cancer types by combining network proximity and GSEA approaches implemented in GPSnet. Importantly, we experimentally validated that two approved non-cancer drugs (ouabain and niclosamide) showed potential anticancer activities in several NSCLC cell lines. Interestingly, ouabain’s targets do not show significant network proximity with SMGs identified from traditional statistics-based genome sequencing analysis in the human interactome (Supplementary Fig. 15), indicating the power of the GPSnet algorithm. In summary, GPSnet offers a powerful network-based framework to identify new therapeutic interventions through uniquely integrating patient sequencing data and the human interactome. If broadly applied, GPSnet could be applied to other diseases as well.

Several potential limitations should be acknowledged. First, previous studies have shown that well-known disease genes often have high data bias in the literature-derived human protein–protein interactome^11^. For this reason, we tested the GPSnet algorithm in the unbiased, systematic Y2H interactome with low literature data bias, and found consistent results based on an integrated human interactome network that we used in this study (Supplementary Fig. 8). For drug target enrichment analysis, we found that known drug targets are enriched in the new disease modules identified by unbiased, systematic Y2H interactome across 4 four cancer types; however, the enrichments are not statistically significant (*p*-value ranges from 0.0667 to 0.0984, permutation test, Supplementary Fig. 16). There are two possible explanations for this finding: (i) literature data bias, and (ii) network data incompleteness. The current Y2H unbiased interactome we used only includes 46,203 PPIs (links) connecting 8072 unique proteins (nodes). We, therefore, used a more comprehensive binary human interactome (including 98,240 PPIs connecting 12,994 unique proteins) published recently^32^. We found that drug targets are significantly enriched in the new disease modules identified from this more comprehensive binary human interactome network (Supplementary Fig. 17). Thus, building a high-quality, comprehensive, unbiased systematic interactome would improve further the performance of GPSnet.

Here, we integrated both GSEA and network proximity approaches (Fig. 2c) for in silico drug repurposing by integrating physical drug-target interactions and functional drug-gene associations with the cancer type-specific disease modules identified by GPSnet into the human interactome. Owing to a lack of large-scale published available experimental data, whether combining GSEA and network proximity synergistically improves the performance of drug repurposing compared to single approaches (GSEA or network proximity) requires further evaluation. Drugs can inhibit or activate protein function (including antagonists vs. agonists), while disease alleles from genetic or genomic studies harbor loss-of-function or gain-of-function phenotypes. For example, an inhibitor that targets loss-of-function disease proteins (e.g., tumor suppressors) often causes adverse effects. Hence, integration of functional genomic assays (e.g., CRISPR-Cas9) or large-scale disease gene expression profiles (e.g., well-defined oncogenes or tumor suppressor genes) along with patient data (e.g., health insurance claims data) for validation and in vitro or in vivo mechanistic studies will improve in silico drug repurposing models in the future^33^. In this study, we experimentally validated that ouabain showed potential antitumor effects in NSCLC cells, consistent with several previous studies^34,35^. Importantly, we demonstrated that ouabain may target HIF1α/LEO1-mediated cell metabolic pathways in NSCLC cells by integrating gene expression, metabolomics data, and network analysis. However, the regulation of HIF1α and LEO1 in NSCLC cell metabolism warrants future studies by several approaches, such as CHIP-seq.

In summary, this study offers a powerful network-based methodology for drug repurposing, and validates experimentally ouabain and niclosamide as potential antitumor agents to treat NSCLC. Importantly, we showed that ouabain potentially targets the HIF1α/LEO1-mediated cell metabolism pathway in NSCLC cells. To the best of our knowledge, GPSnet is a highly innovative network-based methodology that integrates large-scale patient DNA/RNA-seq data with the human protein–protein interactome, enabling accelerated target identification and drug development in cancer and other diseases if broadly applied. In this manner, we can minimize the translational gap between genomic medicine studies and clinical outcomes, a significant goal in network medicine and  precision medicine.

## Methods

### Collection of whole-exome sequencing data

We downloaded the tumor-normal pairwise somatic mutation data for patients from three sources: (1) Elledge Lab website at Harvard University (http://elledgelab.med.harvard.edu/?page_id=689), (2) Sanger website: (ftp://ftp.sanger.ac.uk/pub/cancer/AlexandrovEtAl), and (3) COSMIC: Catalog of Somatic Mutations in Cancer (https://cancer.sanger.ac.uk/cosmic). To reduce redundancy and ensure the quality of somatic mutation data in this study, we only focused on the somatic mutations in TCGA tumor-normal matched samples from the aforementioned three datasets. The RNA-seq (read counts) data for 15 cancer types were downloaded from GDC website (https://portal.gdc.cancer.gov/) for computing co-expression (measured by Pearson Correlation Coefficient) of PPI coding gene pairs.

### Construction of drug-target network

We assembled high-quality physical drug-target interactions for FDA-approved drugs from six commonly used data sources, and defined a physical drug-target interaction using reported binding affinity data: inhibition constant/potency (*K*_i_), dissociation constant (*K*_d_), median effective concentration (EC_50_), or median inhibitory concentration (IC_50_) ≤ 10 µM. Drug-target interactions were acquired from the DrugBank database (v4.3)^36^, the Therapeutic Target Database (TTD, v4.3.02)^37^, and the PharmGKB database^38^. Specifically, bioactivity data of drug-target pairs were collected from three commonly used databases: ChEMBL (v20)^39^, BindingDB^40^, and IUPHAR/BPS Guide to PHARMACOLOGY^41^. After extracting the bioactivity data related to the drugs from the prepared bioactivity databases, only those items meeting the following four criteria were retained: (i) binding affinities, including *K*_i_, *K*_d_, IC_50_, or EC_50_, ≤10 μM; (ii) proteins represented by unique UniProt accession number; (iii) proteins marked as “reviewed” in the UniProt database^42^; and (iv) proteins of human origin.

### Building the human protein–protein interactome

To build the comprehensive human protein–protein interactome as currently available, we assembled 15 commonly used databases with multiple experimental sources of evidence and the in-house systematic human protein–protein interactome: (1) binary PPIs tested by high-throughput yeast-two-hybrid (Y2H) systems in which we combined two publicly available high-quality Y2H datasets^11,43^, and one dataset available from our website: http://ccsb.dana-farber.org/interactome-data.html; (2) kinase-substrate interactions by literature-derived low-throughput and high-throughput experiments from KinomeNetworkX^44^, Human Protein Resource Database (HPRD)^45^, PhosphoNetworks^46,47^, PhosphositePlus^48^, DbPTM 3.0^49^, and Phospho.ELM^50^; (3) carefully literature-curated PPIs identified by affinity purification followed by mass spectrometry (AP-MS), and by literature-derived low-throughput experiments from BioGRID^51^, PINA^52^, HPRD^45^, MINT^53^, IntAct^54^, and InnateDB^55^; (4) high-quality PPIs from protein three-dimensional (3D) structures reported in Instruct^56^; and (5) signaling networks by literature-derived low-throughput experiments as annotated in SignaLink2.0^57^. The genes were mapped to their Entrez ID based on the NCBI database^58^ as well as their official gene symbols based on GeneCards (http://www.genecards.org/). Inferred data, such as evolutionary analysis, gene expression data, and metabolic associations, were excluded. The updated human interactome constructed in this way includes 170,000 protein–protein interactions (PPIs) (edges or links) connecting 15,474 unique proteins (nodes). The unbiased, systematic human interactome^11,43^ was downloaded from our website (https://ccsb.dana-farber.org/interactome-data.html).

### Description of GPSnet

The GPSnet algorithm contains two main components: cancer type-specific disease module identification, and in silico drug repurposing. Under our assumptions, the disease module should be highly connected in the cancer type-specific co-expressed PPI networks derived from RNA-seq data, and the genes in the module should tend to be the highly-mutated genes. We use the random searching method to identify the disease module in GPSnet.

We first set the initial score of each gene *i* in each cancer type as $s_0(i) = \frac{{m(i)}}{{l(i)}}$, where *m*(*i*) is the mutation frequency of gene *i* in the corresponding cancer type, and *l*(*i*) is the cDNA length of gene *i*. In order to eliminate the influence of the somatic mutation data that are sparse, the network smoothing method is used to transmit the score across the whole network.

The random walk with restart process (RWR) is applied to calculate the smoothing gene score. Consider a random walker starting from gene *i*, who will move to a random neighbor with probability $(1 - {\mathrm{\alpha }})$ or returns to gene *i* with probability α at each iterative time step, where ${\mathrm{\alpha }} \in [0\,1]$ is the parameter that drives the restart probability of the random walk process. Here we use *α* = 0.5 to balance the degree bias (Supplementary Figs. 18–21). Denote $\overrightarrow {s_t}$ as the score vector at step *t*, and the propagation process can be described as:

4 $$\overrightarrow {s_{t + 1}} = (1 - \alpha )W\overrightarrow {s_t} + \alpha \overrightarrow {s_0},$$

where $\overrightarrow {s_0}$ is the vector of each gene’s initial score, and *W* is the transfer matrix with $W_{ij} = \frac{1}{{k(j)}}$ if gene *i* interacts with gene *j*, otherwise $W_{ij} = 0$ (with $k(j)$ the number of neighbors of gene *j* in the network). The theoretical solution is:

5 $$\vec s = \alpha \left( {1 - \left( {1 - \alpha } \right)W} \right)^{ - 1}\overrightarrow {s_0}$$

with the *i*th element of $\vec s$ the smoothing score of gene *i*.

The module is defined as a sub-graph within the cancer type-specific co-expressed PPI network, and the score of module *M* is calculated according to Eq. 1, where *m* represents the number of genes in module *M* and *μ* is the average score over the whole gene set for the corresponding cancer type. The following steps are used to perform the random search process to generate a raw module. Initially, a gene is randomly selected as the “seed” gene. We denote Γ_*M*_ as the set of the genes that interact with module *M* in the human interactome. For each gene $i \in \Gamma _M$, we calculate the connectivity significance in the cancer type-specific PPI network by Eq. 2.

For each gene $i \in \Gamma _M$, we calculate the expanded module score if gene *i* is added to the module in Eq. 3. One raw module is obtained by repeating the searching steps until no more genes can be added to the corresponding module. In this study, we built approximately 60,000 raw modules for each cancer type. In this way, each gene in the human interactome was randomly selected approximately five times. We removed the raw modules with less than 10 genes, and collected the top 1% (approximately 300) of modules with the highest module scores, indicating gene confidence by calculating how many times each gene appears in these modules. Finally, we selected the genes with gene confidence values larger than 0.5% and assembled the largest connected component^7^ among these genes (removing isolated nodes) in the cancer type-specific co-expressed PPI network as the final disease module for each cancer type. The detailed description of GPSnet is provided in the Supplementary Note 3 and Supplementary Fig. 4.

### Pharmacogenomics models

We downloaded gene bulk expression profiles and drug response data (defined by IC_50_ value) across cancer cell lines from the GDSC database^17^. We built regression models to predict drug’s IC_50_ value using the LIBSVM^59^ R package with default parameters and linear kernels. Specifically, the GPSnet-identified disease modules with RMA gene expression profiles of cancer cell lines were transformed into a matrix with each column of this matrix denoting a feature vector and each row denoting a cancer cell line from the GDSC database. Finally, the ROC curves were plotted using the R package.

### Gene-set enrichment analysis (GSEA)

We collected drug-gene signatures from the Connectivity Map (CMap, build 02)^23^. The CMap comprises over 7000 gene expression profiles from cultured human cell lines treated with various small bioactive molecules (1309 total), at different concentrations, covering 6100 individual instances. The CMap thus provides a measure of the extent of differential expression for a given probe set. The amplitude (*a*) is defined as follows:

6 $$a = \frac{{t - c}}{{(t + c)/2}},$$

where *t* is the scaled and threshold average difference value for the drug treatment group and *c* is the threshold average difference value for the control group. Thus, *a* = 0 indicates no differential expression, *a* > 0 indicates increased expression (upregulation) upon treatment, and *a* < 0 indicates decreased expression (downregulation) upon treatment. For example, an amplitude of 0.67 represents a two-fold induction^60^. Drug gene signatures with amplitudes >0.67 were defined as upregulated drug-gene pairs, and amplitudes <− 0.67 reflected downregulated drug-gene pairs. We then mapped probe sets to the human genome. In total, we compiled 1,874,674 drug-gene pairs from the CMap, connecting 1309 drugs and 12,768 genes.

For each drug-disease pair, we counted the number of module genes in a particular cancer type identified by GPSnet, and those that are upregulated or downregulated by drug treatments, as well as overlap or mutually exclusive pairs. Here, we used *P* *<* 0.05 as a cutoff to identify significant drug-disease associations (Supplementary Data 5).

### Network proximity

Given *S*, the set of disease proteins, and *T*, the set of drug targets, *d(s,t)*, the closest distance measured by the average shortest path length between node *s* and the nearest disease protein *t* in the human protein–protein interactome, is defined as:

7 $$d(S,T) = \frac{1}{{\left\| T \right\|}}\mathop {\sum }\limits_{t \in T} \,{\mathrm{min}}_{s \in S} d(s,t)$$

To evaluate the significance of the network distance between a drug and a given disease, we constructed a reference distance distribution corresponding to the expected distance between two randomly selected groups of proteins of the same size and degree distribution as the original disease proteins and drug targets in the network. This procedure was repeated 1000 times. The mean $\bar d$ and standard deviation ($\sigma _d$) of the reference distribution were used to calculate a *z*-score ($z_d$) by converting an observed (non-Euclidean) distance to a normalized (non-Euclidean) distance (Supplementary Data 5). The detailed description of network proximity was provided in our recent study^8^.

### Pathway enrichment analysis

We used ClueGO^61^ for enrichment analysis of genes in the canonical KEGG pathways. A hypergeometric test was performed to estimate statistical significance, and all *P* values were adjusted for multiple testing using Bonferroni’s correction (adjusted *P* values).

### Cell culture

All cells were incubated at 37 °C in a humidified incubator with 5% CO_2_. NSCLC cell lines A549, H522, H596, H1975, HCC827, and PC9 were obtained from the American Type Culture Collection (Manassas, VA) and cultured in Roswell Park Memorial Institute (RPMI) 1640 Medium supplemented with 10% fetal bovine serum (FBS, Gibco), and penicillin-streptomycin. Lung normal cell lines MRC5 and WI38 were obtained from the Shanghai Cell Bank of the Chinese Academy of Sciences (Shanghai, China) and maintained in Eagle’s Minimum Essential Medium (EMEM) supplemented with 10% fetal bovine serum (FBS, Gibco) and penicillin-streptomycin (Supplementary Table 1). Cell lines were subjected to a mycoplasma detection test and authenticated by short tandem repeat (STR) profiling.

### Cell viability assay

Cell viability assay was conducted as previously described^62^. In brief, 3000–5000 cells/well were seeded in 96-well plates for 12 h, and then incubated with the indicated compounds for 48 h. Cell viability was detected using CellTiter 96 AQueous One Solution from Promega (Madison, WI), according to the manufacturer’s protocol. IC_50_ values were calculated from dose-response curves using Graphpad Prism 7 (GraphPad Software).

### Colony formation

A549 or H522 cells were seeded into 6-well plates at 1500 cells per well in 2 ml of 1640 medium supplemented with 10% FBS and penicillin-streptomycin. After cell adherence, various concentrations of ouabain were incubated with cells. The medium was changed every 2 days. After 7 days, colonies were fixed in 4% paraformaldehyde and stained with 0.2% crystal violet.

### Western blotting and antibodies

Cell were lysed with buffer containing 100 mM Tris-HCl (pH 7.5),150 mM NaCl, 1 mM EDTA, 0.1% SDS, 1% sodium deoxycholate, and 1% Triton X-100 with protease inhibitors (Roche, San Francisco, CA) and phosphatase inhibitors (Santa Cruz, Dallas, TX). Protein concentration was determined using the bicinchoninic acid assay (BCA) (Thermo Fisher, Waltham, MA). Equal amounts of proteins samples (approximately 50 μg) were run on SDS-polyacrylamide gel electrophoresis and transferred to nitrocellulose membranes (Millipore, Burlington, MA). The membranes were blocked in TBST (10 mM Tris-HCl pH = 8, 150 mM NaCl, 0.1% Tween) with 5% BSA and probed with primary antibodies and corresponding fluorescence-conjugated secondary antibodies. Antibodies (Supplementary Table 2) used included: anti-GAPDH (Catalog No.: AB0037, 1:10,000 dilution) and anti-HIF1α (Catalog No.: CY5197, 1:1000 dilution), purchased from Abways Technology (Shanghai, China); and anti-LEO1 (Catalog No.: ab75721, 1:100 dilution), purchased from Abcam (Cambridge, MA). Uncropped images of Figs. 6c, 7d are included as Supplementary Figs. 22 and 23.

### Real-time quantitative PCR (RT-qPCR)

Total RNA was isolated using the Trizol reagent (Invitrogen, Carlsbad, CA) according to the manufacturer’s protocol. cDNA synthesis was performed using the ReverTra Ace qPCR RT Master Mix (TOYOBO, OSAKA, Japan). qPCR reactions were conducted using SYBR Green Real-Time PCR Master Mixes (Thermo Fisher, Waltham, MA) on CFX-96TM (Bio-Rad, Hercules, CA). The amount of each gene was detected and normalized by the amount of GAPDH. The Ct values were generated using the standard curve method. The sequences of primers used in RT-qPCR experiments (Supplementary Table 3) are as follows: *HIF1A*: Forward primer: 5′-ACTCAGGACACAGATTTAGACTTG-3′. Reverse primer: 5′-TGGCATTAGCAGTAGGTTCTTG-3′.

*LEO1*: Forward primer: 5′-AGAAGCGGATAGTGACACTGAGGT-3′.

Reverse primer: 5′-TTCATCAACAGGCTGTCCTGGAGT-3′. pLKO.1-puro vectors encoding shRNA targeting HIF1A were purchased from Synbio Technologies. shHIF1A-1 sequence: CCGGGTGATGAAAGAATTACCGAATCTCGAGATTCGGTAATTCTTTCATCACTTTTT; shHIF1A-2 sequence: CCGGTGCTCTTTGTGGTTGGATCTACTCGAGTAGATCCAACCACAAAGAGCATTTTT. HIF1A knockdown was confirmed by a RT-qPCR.

### Transcription factor network analysis

We collected the 719 putative reported HIF1A transcription factor targets (Supplementary Data 6) from two previous studies^63,64^. To inspect the potential function of HIF1A transcription factor in LUAD, we calculated the differential expression of these 719 genes (Supplementary Data 6) by utilizing the RNA-seq read count data in LUAD patient’s tumor samples compared to the matched normal samples from TCGA using DESeq2^65^. We used the adjusted *P*-value less than 0.05 to define the differentially expressed genes.

### Correlation between metabolite abundance and gene expression

We collected [^13^C]serine or [^13^C]glycine abundance tested in ~70 NSCLC cell lines from a previous study^30^. We next collected genome-wide gene expression profiles on NSCLC cell lines from the CCLE database^29^. The correlation between metabolite abundance and gene expression level (Supplementary Data 6) was computed by Pearson correlation coefficient measure, and *P*-values were computed by the *F*-statistics using the R platform (v3.01, http://www.r-project.org/).

### Tissue-specific subnetwork analysis

We downloaded the RNA-seq data (RPKM value) of 32 tissues from GTEx V6 release (https://gtexportal.org/home/). For each tissue (e.g., lung), we regarded those genes with RPKM ≥1 in more than 80% of samples as tissue-expressed genes and the remaining genes as tissue-unexpressed. To quantify the expression significance of tissue-expressed gene *i* in tissue *t*, we calculated the average expression *E*(*i*) and the standard deviation $\delta _E(i)$ of a gene’s expression across all considered tissues. The significance of gene expression in tissue *t* is defined as $z_E(i,t) = (E(i,t) - E(i))/\delta _E(i)s$ as described previously^8^. For LUAD, we built a lung-specific protein–protein interaction network by comparing genome-wide expression profiles of lung to 31 other different tissues from GTEx.

### Survival analysis

We downloaded the microarray data and survival profiles on 226 primary human stage I–II lung adenocarcinomas (GSE31210)^66^. Patients were grouped into the top 50% lowly expressed (blue) versus top 50% highly expressed (red) groups based on the normalized expression level. The *P*-value for Kaplan–Meier survival analysis was determined using a log-rank test in the GraphPad Prism 7 software.

### Statistical analysis

The data shown in the study were obtained from at least three independent experiments and all data in different experimental groups were expressed as the mean ± the standard error of the mean (SEM). Differences between the two groups were analyzed by Student’s *t*-test and *P* values as indicated in the figure legends. *P < 0.05 was considered statistically significant. Statistical analyses were conducted using GraphPad Prism 7 software.

### Reporting summary

Further information on research design is available in the Nature Research Reporting Summary linked to this article.

## Supplementary information

Supplementary Information

Description of Additional Supplementary Files

Supplementary Data 1

Supplementary Data 2

Supplementary Data 3

Supplementary Data 4

Supplementary Data 5

Supplementary Data 6

Reporting Summary

## Data Availability

The unpublished binary human protein–protein interactions can be accessed at http://ccsb.dana-farber.org/interactome-data.html. Data supporting the findings of this study are available within Supplementary Data Files 1–6, and additional data are available from the corresponding author upon reasonable request.

## Code availability

The code for GPSnet analysis is available at https://github.com/ChengF-Lab/GPSnet. The other codes written for and used in this study are available from the corresponding author upon reasonable request.

## References

[1] International Human Genome Sequencing Consortium. (2004). Finishing the euchromatic sequence of the human genome. Nature.

[2] Dugger SA, Platt A, Goldstein DB (2018). Drug development in the era of precision medicine. Nat. Rev. Drug Discov..

[3] Antman EM, Loscalzo J (2016). Precision medicine in cardiology. Nat. Rev. Cardiol..

[4] Mullard A (2017). Nine paths to PCSK9 inhibition. Nat. Rev. Drug Discov..

[5] Sahni N (2015). Widespread macromolecular interaction perturbations in human genetic disorders. Cell.

[6] Jongen-Lavrencic M (2018). Molecular minimal residual disease in acute myeloid leukemia. N. Engl. J. Med..

[7] Menche J (2015). Disease networks. Uncovering disease-disease relationships through the incomplete interactome. Science.

[8] Cheng F (2018). Network-based approach to prediction and population-based validation of in silico drug repurposing. Nat. Commun..

[9] Wang RS, Loscalzo J (2018). Network-based disease module discovery by a novel seed connector algorithm with pathobiological implications. J. Mol. Biol..

[10] Cheng F, Kovacs IA, Barabasi AL (2019). Network-based prediction of drug combinations. Nat. Commun..

[11] Rolland T (2014). A proteome-scale map of the human interactome network. Cell.

[12] Cheng F (2014). Studying tumorigenesis through network evolution and somatic mutational perturbations in the cancer interactome. Mol. Biol. Evol..

[13] Leiserson MD (2015). Pan-cancer network analysis identifies combinations of rare somatic mutations across pathways and protein complexes. Nat. Genet..

[14] Hofree M, Shen JP, Carter H, Gross A, Ideker T (2013). Network-based stratification of tumor mutations. Nat. Methods.

[15] Ghiassian SD, Menche J, Barabasi AL (2015). A DIseAse MOdule Detection (DIAMOnD) algorithm derived from a systematic analysis of connectivity patterns of disease proteins in the human interactome. PLoS Comput. Biol..

[16] Futreal PA (2004). A census of human cancer genes. Nat. Rev. Cancer.

[17] Yang W (2012). Genomics of drug sensitivity in cancer (GDSC): a resource for therapeutic biomarker discovery in cancer cells. Nucleic Acids Res..

[18] Fong PC (2009). Inhibition of poly(ADP-ribose) polymerase in tumors from BRCA mutation carriers. N. Engl. J. Med..

[19] Bollag G (2012). Vemurafenib: the first drug approved for BRAF-mutant cancer. Nat. Rev. Drug Discov..

[20] Lim HY (2018). Phase II studies with refametinib or refametinib plus sorafenib in patients with RAS-mutated hepatocellular carcinoma. Clin. Cancer Res..

[21] Okada Y (2014). Genetics of rheumatoid arthritis contributes to biology and drug discovery. Nature.

[22] Subramanian A (2005). Gene set enrichment analysis: a knowledge-based approach for interpreting genome-wide expression profiles. Proc. Natl Acad. Sci. USA.

[23] Lamb J (2006). The Connectivity Map: using gene-expression signatures to connect small molecules, genes, and disease. Science.

[24] Zhang J (2007). SRC-family kinases are activated in non-small cell lung cancer and promote the survival of epidermal growth factor receptor-dependent cell lines. Am. J. Pathol..

[25] Siddiqui N, Sonenberg N (2015). Signalling to eIF4E in cancer. Biochem. Soc. Trans..

[26] Semenza GL (2003). Targeting HIF-1 for cancer therapy. Nat. Rev. Cancer.

[27] Cancer Genome Atlas Research, N. (2014). Comprehensive molecular profiling of lung adenocarcinoma. Nature.

[28] Zhao Y (2015). SoNar, a highly responsive NAD+/NADH sensor, allows high-throughput metabolic screening of anti-tumor agents. Cell Metab..

[29] Barretina J (2012). The cancer cell line encyclopedia enables predictive modelling of anticancer drug sensitivity. Nature.

[30] DeNicola GM (2015). NRF2 regulates serine biosynthesis in non-small cell lung cancer. Nat. Genet..

[31] Chong PS (2014). LEO1 is regulated by PRL-3 and mediates its oncogenic properties in acute myelogenous leukemia. Cancer Res..

[32] Meyer MJ (2018). Interactome INSIDER: a structural interactome browser for genomic studies. Nat. Methods.

[33] Cheng F, Zhao J, Fooksa M, Zhao Z (2016). A network-based drug repositioning infrastructure for precision cancer medicine through targeting significantly mutated genes in the human cancer genomes. J. Am. Med. Inform. Assoc..

[34] Pongrakhananon V, Chunhacha P, Chanvorachote P (2013). Ouabain suppresses the migratory behavior of lung cancer cells. PLoS One.

[35] Liu N (2013). Inhibition of cell migration by ouabain in the A549 human lung cancer cell line. Oncol. Lett..

[36] Law V (2014). DrugBank 4.0: shedding new light on drug metabolism. Nucleic Acids Res..

[37] Zhu F (2012). Therapeutic target database update 2012: a resource for facilitating target-oriented drug discovery. Nucleic Acids Res..

[38] Hernandez-Boussard T (2008). The pharmacogenetics and pharmacogenomics knowledge base: accentuating the knowledge. Nucleic Acids Res..

[39] Gaulton A (2012). ChEMBL: a large-scale bioactivity database for drug discovery. Nucleic Acids Res..

[40] Liu TQ, Lin YM, Wen X, Jorissen RN, Gilson MK (2007). BindingDB: a web-accessible database of experimentally determined protein-ligand binding affinities. Nucleic Acids Res..

[41] Pawson AJ (2014). The IUPHAR/BPS Guide to PHARMACOLOGY: an expert-driven knowledgebase of drug targets and their ligands. Nucleic Acids Res..

[42] Apweiler R (2004). UniProt: the Universal Protein knowledgebase. Nucleic Acids Res..

[43] Luck, K. et al. A reference map of the human protein interactome. Preprint at *bioRxiv* 10.1101/605451 (2019).

[44] Cheng F, Jia P, Wang Q, Zhao Z (2014). Quantitative network mapping of the human kinome interactome reveals new clues for rational kinase inhibitor discovery and individualized cancer therapy. Oncotarget.

[45] Peri S (2004). Human protein reference database as a discovery resource for proteomics. Nucleic Acids Res..

[46] Newman RH (2013). Construction of human activity-based phosphorylation networks. Mol. Syst. Biol..

[47] Hu J (2014). PhosphoNetworks: a database for human phosphorylation networks. Bioinformatics.

[48] Hornbeck PV (2015). PhosphoSitePlus, 2014: mutations, PTMs and recalibrations. Nucleic Acids Res..

[49] Lu CT (2013). DbPTM 3.0: an informative resource for investigating substrate site specificity and functional association of protein post-translational modifications. Nucleic Acids Res..

[50] Dinkel H (2011). Phospho.ELM: a database of phosphorylation sites–update 2011. Nucleic Acids Res..

[51] Chatr-Aryamontri A (2015). The BioGRID interaction database: 2015 update. Nucleic Acids Res..

[52] Cowley MJ (2012). PINA v2.0: mining interactome modules. Nucleic Acids Res..

[53] Licata L (2012). MINT, the molecular interaction database: 2012 update. Nucleic Acids Res..

[54] Orchard S (2014). The MIntAct project–IntAct as a common curation platform for 11 molecular interaction databases. Nucleic Acids Res..

[55] Breuer K (2013). InnateDB: systems biology of innate immunity and beyond–recent updates and continuing curation. Nucleic Acids Res..

[56] Meyer MJ, Das J, Wang X, Yu H (2013). INstruct: a database of high-quality 3D structurally resolved protein interactome networks. Bioinformatics.

[57] Fazekas D (2013). SignaLink 2—a signaling pathway resource with multi-layered regulatory networks. BMC Syst. Biol..

[58] Coordinators NR (2016). Database resources of the National Center for Biotechnology Information. Nucleic Acids Res..

[59] Chang C-C (2011). LIBSVM: a library for support vector machines. ACM Trans. Intel. Syst. Technol..

[60] Cheng F (2016). Systems biology-based investigation of cellular antiviral drug targets identified by gene-trap insertional mutagenesis. PLoS Comput. Biol..

[61] Bindea G (2009). ClueGO: a Cytoscape plug-in to decipher functionally grouped gene ontology and pathway annotation networks. Bioinformatics.

[62] Jiang X (2018). Repurposing sertraline sensitizes non-small cell lung cancer cells to erlotinib by inducing autophagy. JCI Insight.

[63] Xia X, Kung AL (2009). Preferential binding of HIF-1 to transcriptionally active loci determines cell-type specific response to hypoxia. Genome Biol..

[64] Bovolenta LA, Acencio ML, Lemke N (2012). HTRIdb: an open-access database for experimentally verified human transcriptional regulation interactions. BMC Genom..

[65] Love MI, Huber W, Anders S (2014). Moderated estimation of fold change and dispersion for RNA-seq data with DESeq2. Genome Biol..

[66] Okayama H (2012). Identification of genes upregulated in ALK-positive and EGFR/KRAS/ALK-negative lung adenocarcinomas. Cancer Res..

