# Supplementary material for: A genome-wide positioning systems network algorithm for in silico drug repurposing
Source: Nat Commun. 2019 Aug 2;10:3476. doi: 10.1038/s41467-019-10744-6 (PMC6677722; doi:10.1038/s41467-019-10744-6)
Supplement: Supplementary file 1 — Supplementary Information [file 41467_2019_10744_MOESM1_ESM.pdf]

Supplementary Information

Title: A Genome-wide Positioning Systems Network Algorithm for *in silico* Drug Repurposing

Cheng et al., *Nature Communications* 2019

*To whom correspondence should be addressed:

Joseph Loscalzo, M.D., Ph.D.
Brigham and Women's Hospital
75 Francis St.
Boston, MA 02115
Phone: 617-732-6340; fax: 617-732-6439;
Email: jloscalzo@rics.bwh.harvard.edu

Table of contents

Supplementary Information files contain Supplementary Notes 1-3, 3 Supplementary Tables, 23 Supplementary Figures, and Supplementary References

Supplementary Note 1: Modularity of highly Mutated Genes in the unbiased, systematic human protein-protein interactome

Disease proteins are not scattered randomly in the human protein-protein interactome, but form one or several connected subgraphs, defining the disease module[1]. Previous studies have suggested the literature bias for the human protein-protein interactome, with well-studied proteins often having high connectivity (degree) in the literature-derived data[2]. To inspect the potential literature biased, we utilized the unbiased, systematic human protein-protein interactome identified by (unbiased) yeast two-hybrid (Y2H) assays (see Methods). We found that the significantly mutated genes or highly mutated genes form significant modules in this unbiased interactome, as well (Supplementary **Fig. 1**), suggesting low literature data bias.

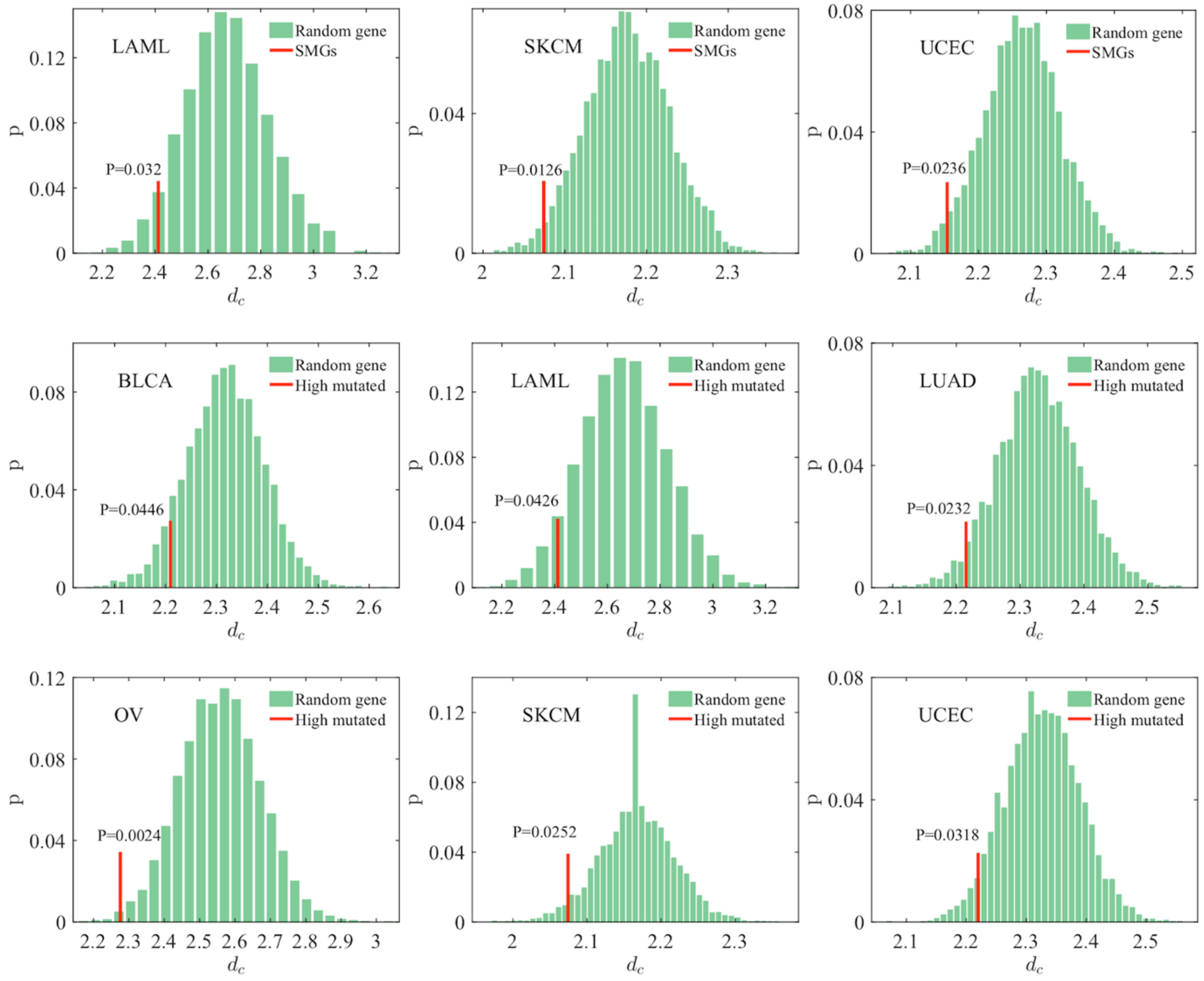

Supplementary **Fig. 1.** Proof-of-concept of disease module for mutant genes derived from patient-specific DNA sequencing data in the unbiased, comprehensive human protein-protein interactome. Both significantly mutated genes (SMGs, Supplementary **Data 1**) identified by statistical approaches and highly mutated genes ranked by mutation frequency have the closest network distance compared to random genes by degree-control randomization in this unbiased, comprehensive human protein-protein interactome (https://ccsb.dana-farber.org/interactome-data.html).

Supplementary Note 2: Genes are highly mutated in network modules from the co-expressed protein-protein interaction network

We define a network module based on the RNA-seq data and PPI network. For each cancer type, we computed the Pearson Correlation Coefficient ($PCC(i,j)$) for each PPI coding gene pair between gene *i* and gene *j,* and we only retained the significantly co-expressed pairs (p-value less than 0.05, F-statistic) for both tumor samples ($PCC(i,j)^T$) and normal samples ($PCC(i,j)^N$) based on RNA-seq data. We used $|PCC(i,j)^T - PCC(i,j)^N| > 0.7$ as a cutoff to select the differentially co-expressed protein-protein interactions, and defined the largest connected component[1] as the network module for the corresponding cancer type. We found that genes in the network modules identified from the RNA-seq data-based co-expressed PPI network are more likely mutated as shown in **Supplementary Fig. 2**. Furthermore, known significantly mutated genes (SMGs) are significantly enriched in the network modules identified from RNA-seq data-based co-expressed PPI networks across four selected cancer types, as well (**Supplementary Fig. 3**). These observations support the hypothesis that highly mutated genes are more differentially co-expressed in the human interactome.

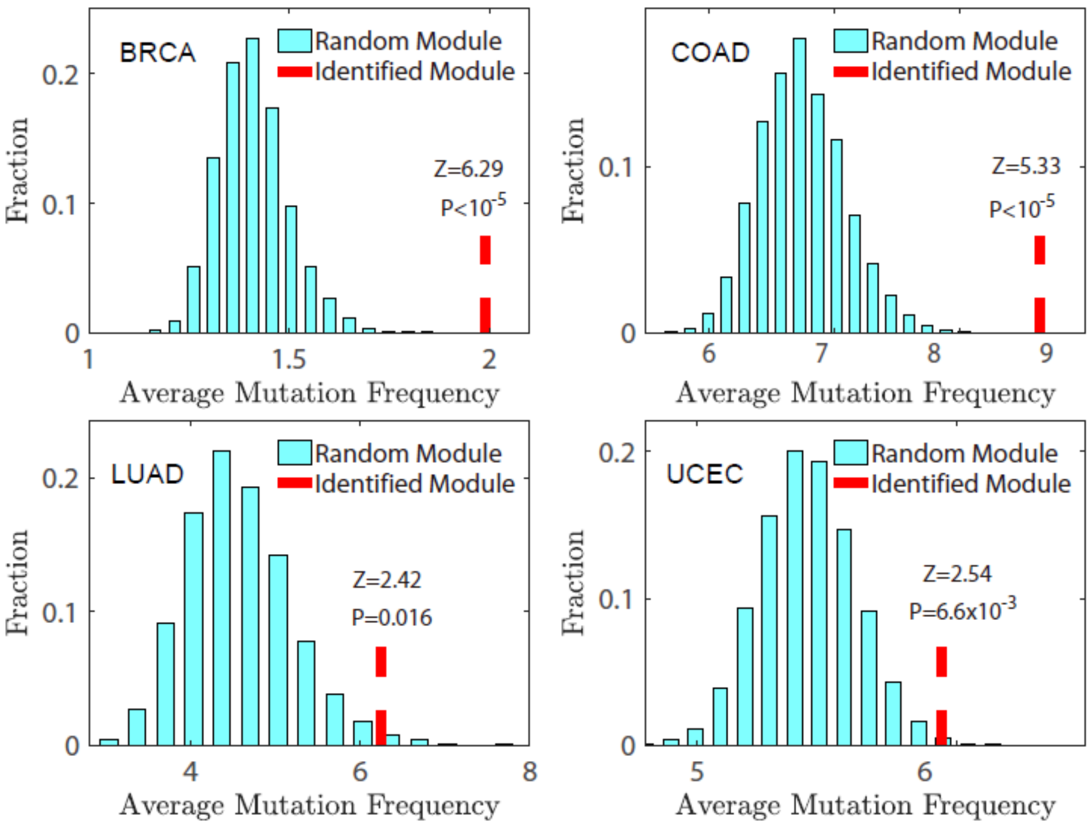

Supplementary **Fig. 2.** Normalized mutation frequency (average mutation frequency) of genes in the network modules identified from RNA-seq data only (red line) compared to the same number of randomly selected genes (cyan histogram) with similar degree (connectivity) distribution in the human protein-protein interactome in four selected cancer types: invasive breast carcinoma (BRCA), lung adenocarcinoma (LUAD), colon adenocarcinoma (COAD), and uterine corpus endometrial carcinoma (UCEC).

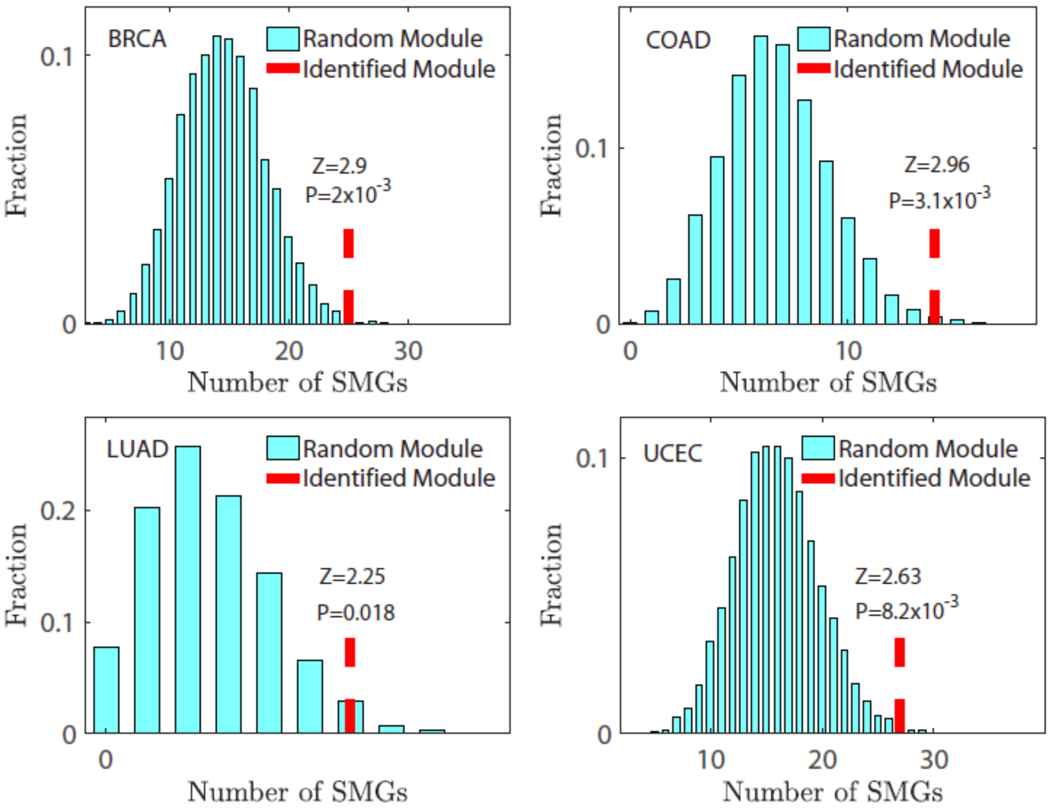

Supplementary **Fig. 3.** Known significantly mutated gene (SMG) enrichment analysis in the new disease modules identified from RNA-seq data only (red line) compared to the same number of randomly selected genes (cyan histogram) with similar degree (connectivity) distribution in the human protein-protein interactome in four selected cancer types: invasive breast carcinoma (BRCA), lung adenocarcinoma (LUAD), colon adenocarcinoma (COAD), and uterine corpus endometrial carcinoma (UCEC).

Supplementary Note 3: Methodology and Detailed Description of GPSnet

Here we present GPSnet, an integrated, network-based methodology for patient-specific disease module identification and *in silico* drug repurposing. Supplementary **Fig. 4** illustrates the pipeline of the GPSnet algorithm.

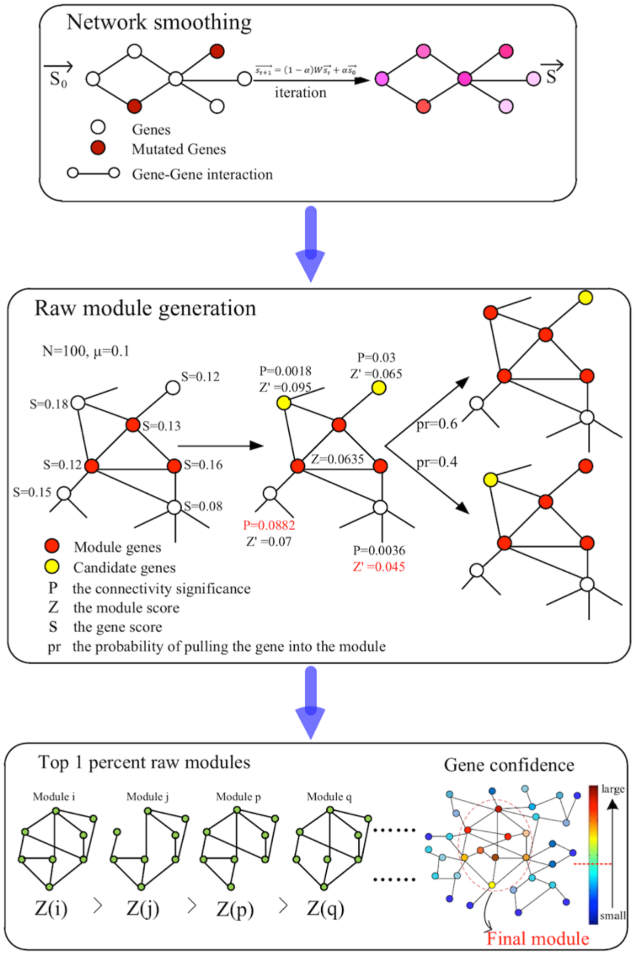

Supplementary **Fig. 4.** A diagram illustrating the GPSnet methodology as described as below.

We aim to find the hyper-mutated module for each caner type, where the number of mutations of the genes in the module is significantly larger than random modules. We set the initial score of each gene (*i*) in each cancer type as $s_0(i) = \frac{m(i)}{l(i)}$, where $m(i)$ is the number of the mutation of gene *i* in the corresponding cancer type, and $l(i)$ is the cDNA length of gene *i*. In order to eliminate the influence of the sparse somatic

mutations, the network smoothing method is used to transmit the score across the whole human protein-protein interactome network.

The random walk with restart process (*RWR*) is applied to calculate the smoothing gene score. Consider a random walker starting from gene *i*, who will move to a random neighbor with probability $(1-\alpha)$ or will return to gene *i* with probability α at each iterative time step, where $\alpha \in [0\ 1]$ is the parameter that drives the restart probability of the random walk process. The *RWR* process is run until a steady-state is reached. We denote $\overrightarrow{s_t}$ as the score vector at iterative step t, and the resulting propagation process can be described as

$$\overrightarrow{s_{t+1}} = (1-\alpha)W\overrightarrow{s_t} + \alpha\overrightarrow{s_0} \qquad (1)$$

where, $\overrightarrow{s_0}$ is the vector of each gene's initial score, and W is the transfer matrix with $W_{ij} = \frac{1}{k(j)}$ if gene *i* interacts with gene *j*, and $W_{ij} = 0$ otherwise ($k(j)$ is the degree of gene *j*). The theoretical solution is to this equation

$$\vec{s} = \alpha(1-(1-\alpha)\mathrm{W})^{-1}\overrightarrow{s_0} \qquad (2)$$

where the *i*-th element of $\vec{s}$ is the smoothing score of gene *i*. The module is defined as a sub-graph within the network of each cancer type, and the score of the module *M* is $Z_M = \frac{\sum_{i\in M}(s(i)-\mu)}{\sqrt{m}}$, where *m* is the number of genes in module *M* and μ is the average score over the whole gene set for the corresponding cancer type. We denote Γ_M as the set of the genes that interact with module *M*.

The following steps are used for the random searching process needed to generate the module.

(1) Initially, a random gene is selected as the "seed" module.
(2) For each gene $i \in \Gamma_M$, we calculate the connectivity significance as follows (extended from the hypergeometric distribution):

$$\mathrm{P(i)} = \sum_{k=k_m}^{k_i} \frac{\binom{m}{k}\binom{N-m}{k_i-k}}{\binom{N}{k_i}} \qquad (3)$$

where k_i is the degree of gene *i*, m is the number of genes in the module, k_m is the number of gene *i*'s neighbors that belong to the module and N is the total number of the gene set.
(3) For each gene $i \in \Gamma_M$, we calculate the expanded module score if gene *i* is added to the module as follows:

$$Z_{m+1}(i) = \frac{(s(i)-\mu)+\sum_{j\in M}(s(j)-\mu)}{\sqrt{m+1}} \qquad (4)$$

(4) The candidate gene *i* that would add to the module should satisfy two constraints, $P(i) < 0.05$ and $Z_{m+1}(i) > Z_m$. Gene *i* will be included in the growing module with probability $\frac{s(i)}{\sum_{i \in cg} s(i)}$ in this time step, where *cg* is the set of the candidate genes.

(5) Steps (2)-(4) are repeated until no more genes can be added.

Repeating the above steps, we obtain a set of modules. We rank the modules according to the descending order of their final score. The gene confidence is calculated as the number of times that the genes appear in the top 1 percent of modules. Genes are then sorted in descending order of the confidence score, and the top *L* genes are considered as the final consensus module (Supplementary **Data 2**).

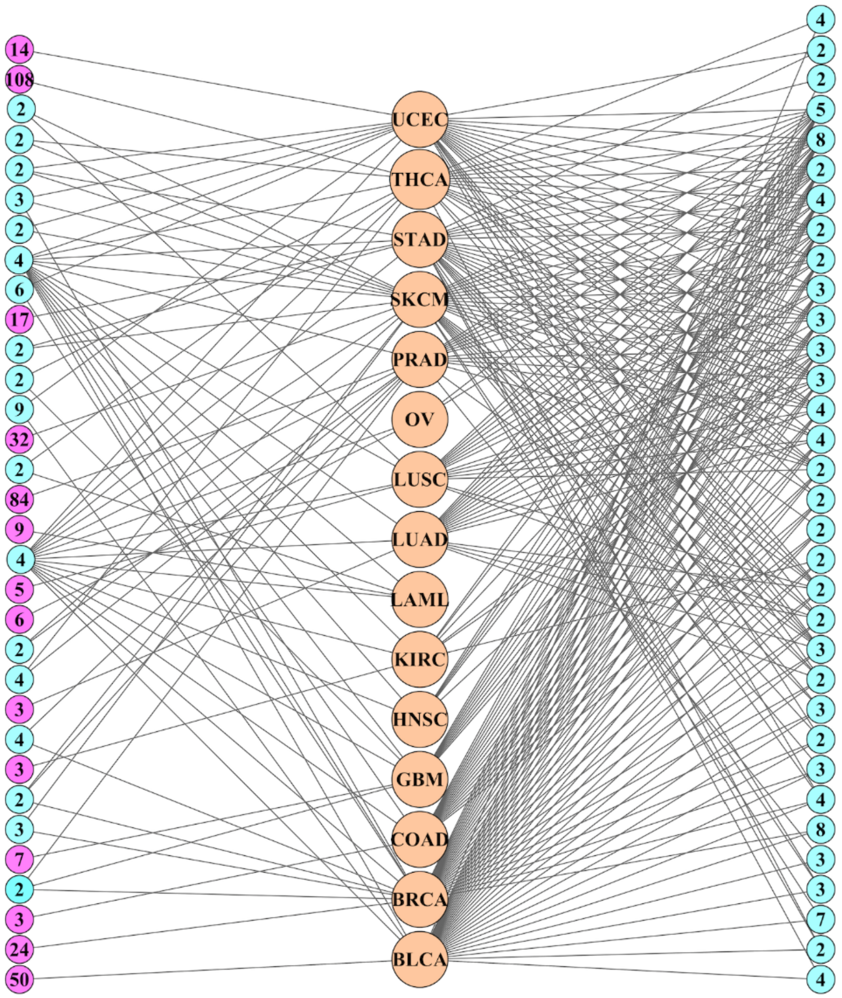

Supplementary **Fig. 5.** Network plot of the number of cancer-specific and shared genes between the disease modules across 15 cancer types. The numbers in the cyan circles denote the numbers of common (shared or overlapped) genes among the corresponding cancer types. The purple circles represent the number of unique genes for specific cancer types.

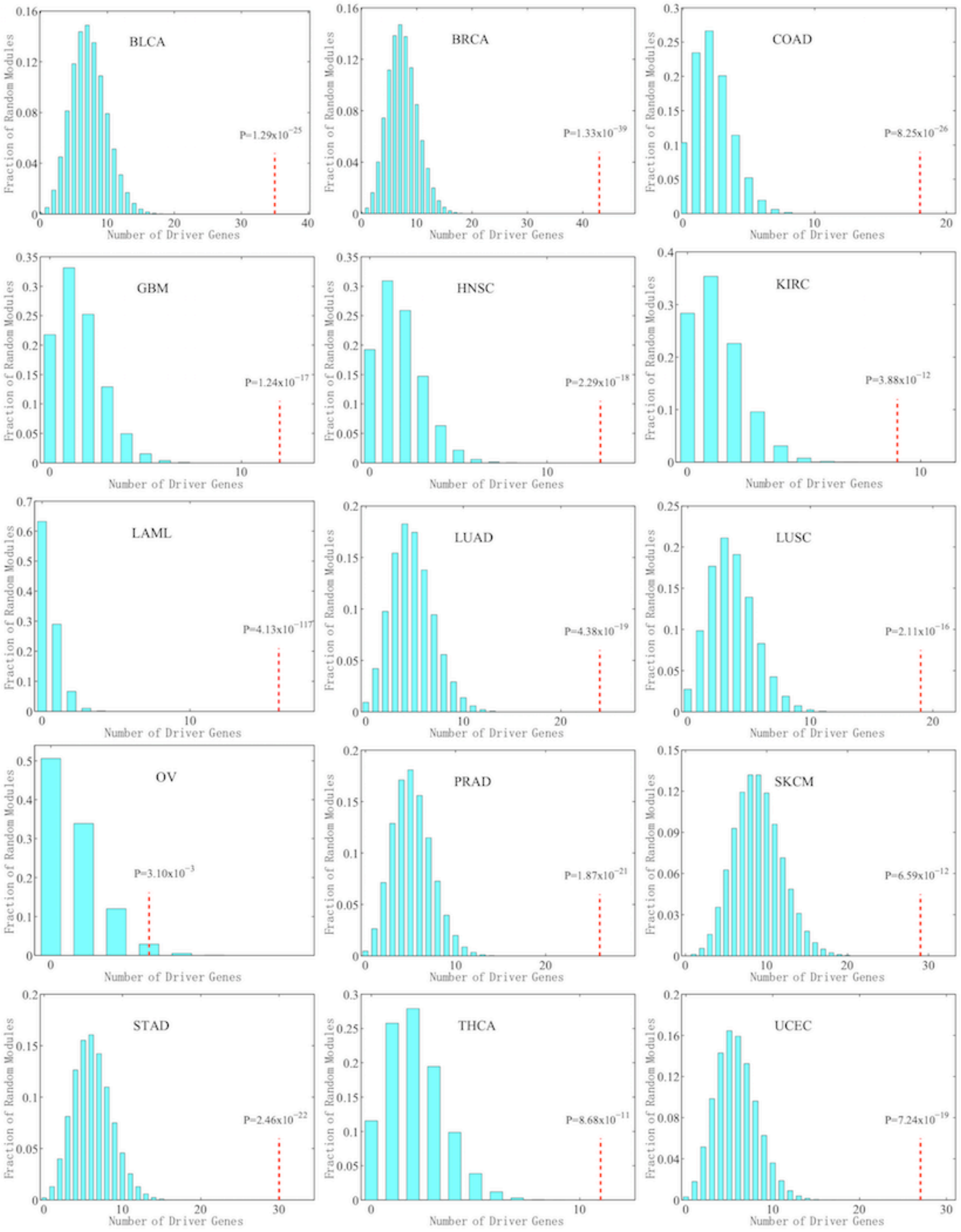

Supplementary **Fig. 6.** Known cancer driver genes (named significantly mutated genes, Supplementary **Data 1**) are appreciably enriched in cancer type-specific disease modules across 15 cancer types. Disease modules were identified by GPSnet when α = 0.5 to balance the degree bias. The significantly mutated genes were collected from TCGA projects as described in previous studies[3,4].

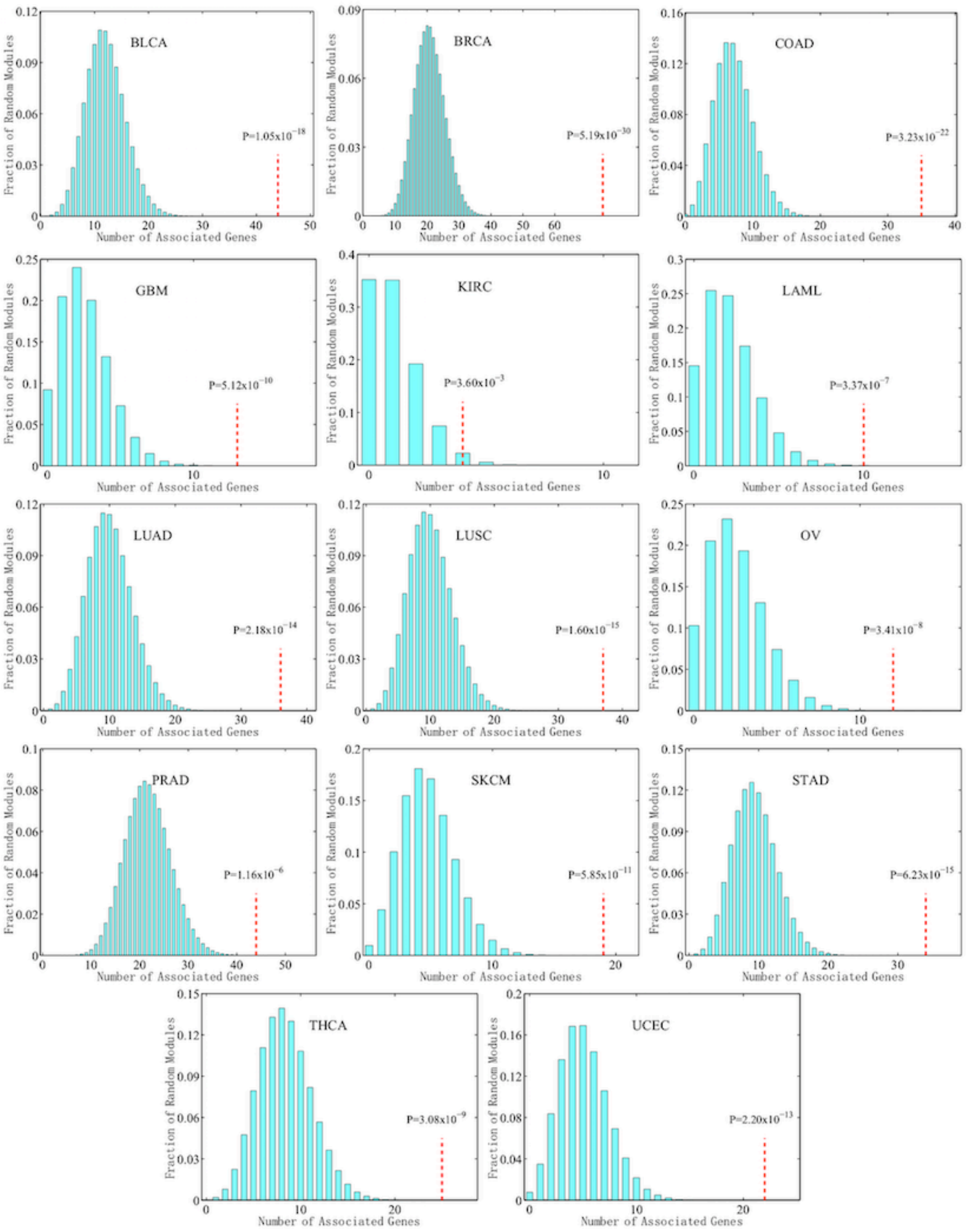

Supplementary **Fig. 7.** Known cancer-associated genes (Supplementary **Data 3**) are appreciably enriched in cancer type-specific disease modules across 14 cancer types. Disease modules were identified by GPSnet when $\alpha = 0.5$ to balance the degree bias. HNSC was excluded for validation owing to lack of known cancer-associated genes from publicly available databases. Known cancer-associated genes were collected from four public databases: the Online Mendelian Inheritance in Man (OMIM) database[5], HuGE Navigator[6], PharmGKB[7], and Comparative Toxicogenomics Database (CTD)[8], as described in our recent study[9].

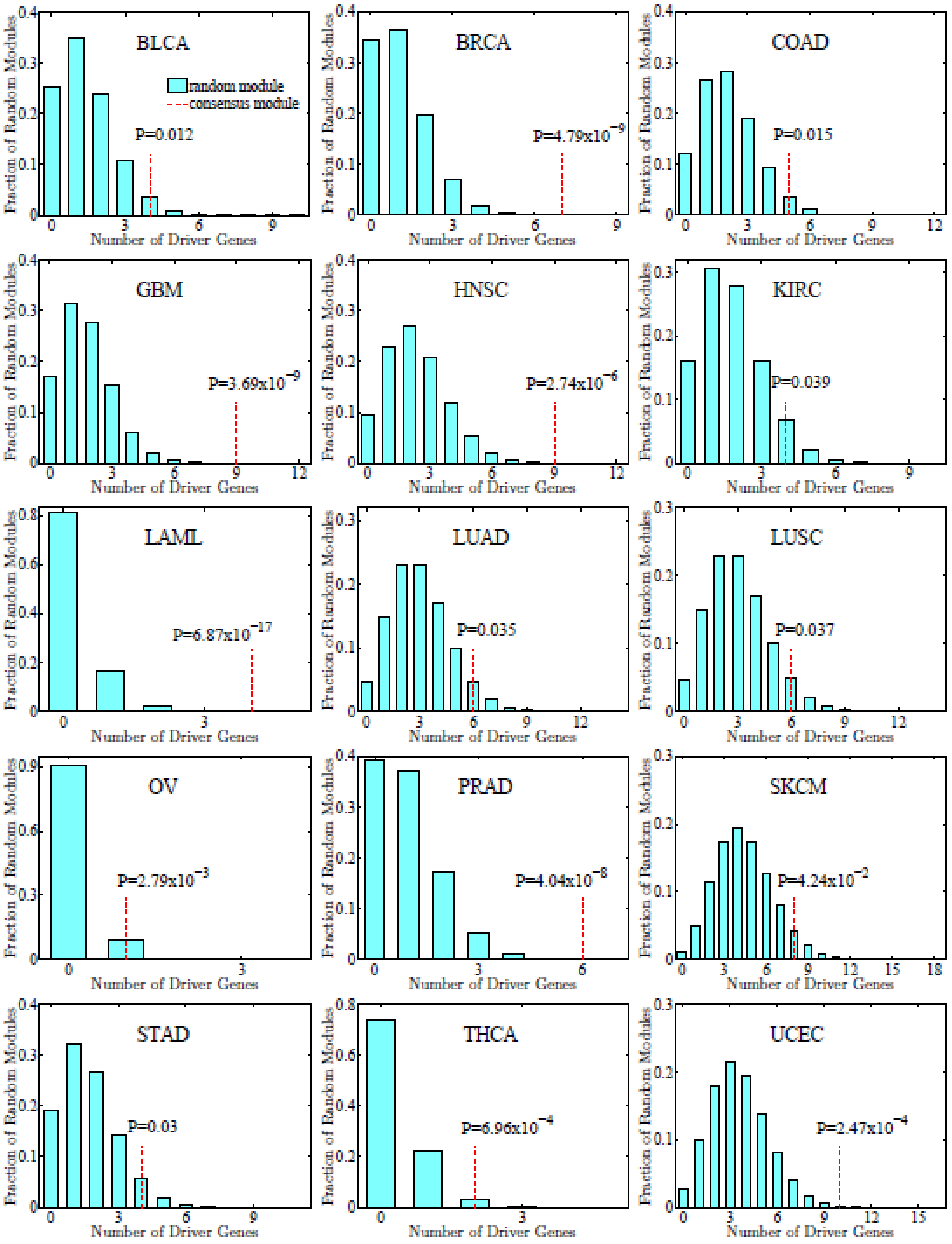

Supplementary **Fig. 8.** Known cancer driver genes (named significantly mutated genes, Supplementary **Data 1**) are appreciably enriched in patient-specific disease modules across 15 cancer types. Disease modules were identified by GPSnet from the unbiased, comprehensive human protein-protein interactome when α = 0.5 (Eq. 4 and 5) to balance the degree bias.

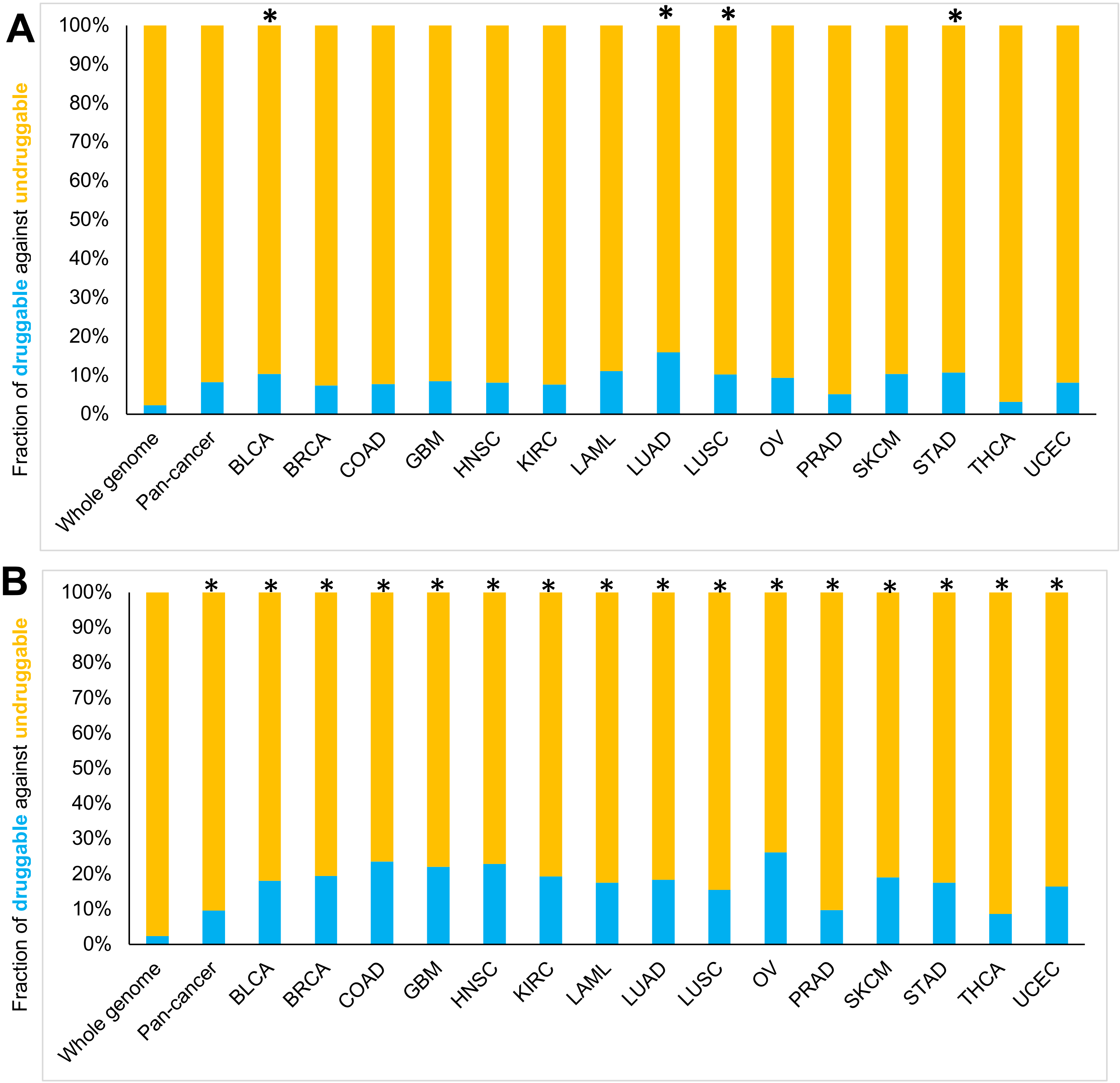

Supplementary **Fig. 9.** Drug target enrichment analysis. Drug target enrichment analysis for (**A**) significantly mutated genes identified by statistics-based approaches alone (red) and (**B**) patient-specific disease module genes (orange) identified by GPSnet algorithm from the human interactome. The distribution of druggable gene products (proteins that

can be targeted by known approved or experimental drugs, see Methods) versus undruggable targets (proteins that cannot be targeted by any available approved or experimental drugs) in patient-specific disease modules across 15 cancer types. * P-value < 1.0×10^{-5}: Gene products in patient-specific disease modules are more likely to be targeted by available approved or experimental drugs compared to whole genome by Fisher's exact test.

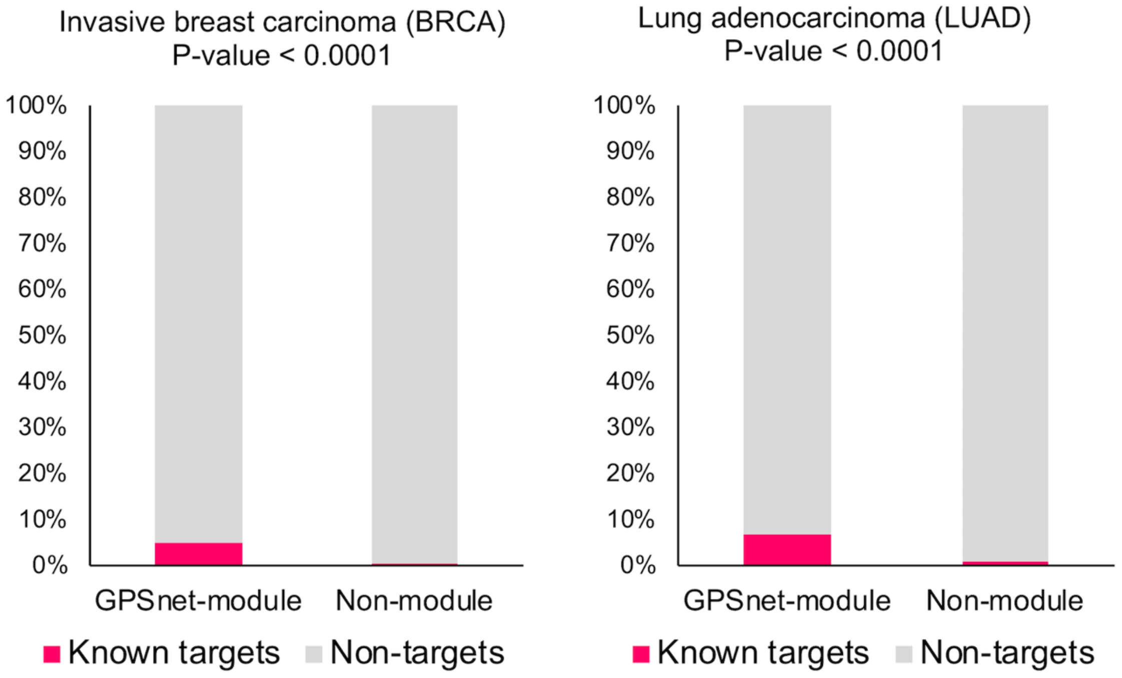

Supplementary **Fig. 10.** Drug target enrichment analysis of GPSnet-identified disease modules in two specific cancer types: invasive breast carcinoma (BRCA) and lung adenocarcinoma (LUAD). We collected the FDA-approved cancer type-specific drugs from the NCI drug database (https://www.cancer.gov/about-cancer/treatment/drugs/) for all 15 cancer types/subtypes (Supplementary **Data 4**). We found that the GPSnet-identified disease module contains drug targets of drugs known to treat this cancer, while the non-module did not. P-value was computed by Fisher's exact test.

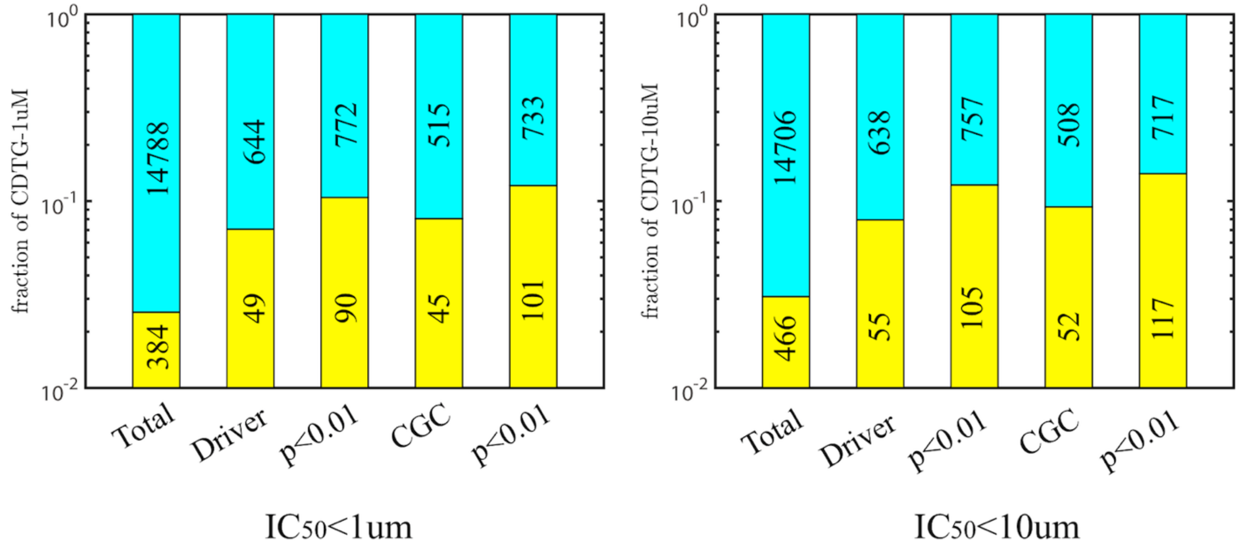

Supplementary **Fig. 11.** Druggable human interactome analysis. We found that the significant neighbors of cancer driver genes (significantly mutated genes, Supplementary **Data 1**) or experimentally validated cancer genes (Cancer Gene Census [CGC] collected from COSMIC database [https://cancer.sanger.ac.uk/census]) are more likely to be targeted by approved drugs compared to driver genes or CGC gene products alone. We identified significant neighbors of proteins from the human protein-protein interactome via the DIAMOnD algorithm[10]. Herein, we tested two types of drug targets: (a) drug-protein binding affinity (IC_{50}) less than 1 μM, and (b) drug-protein binding affinity (IC_{50}) less than 10 μM.

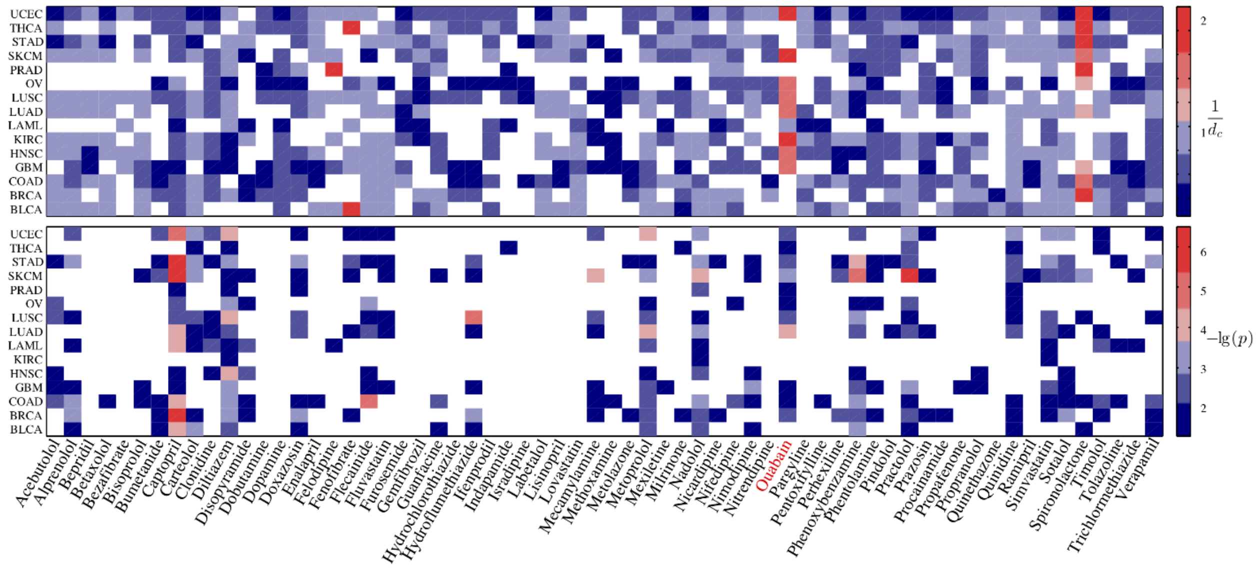

Supplementary **Fig. 12.** A heatmap of computationally predicted anticancer indications for approved cardiovascular drugs (defined by first-level Anatomical Therapeutic Chemical Classification codes) across 15 cancer types, identified by both network proximity ($1/d_c$) and gene-set enrichment analysis (-log(p)) approaches (Supplementary **Data 5**). The detailed data are provided in Supplementary **Data 5**.

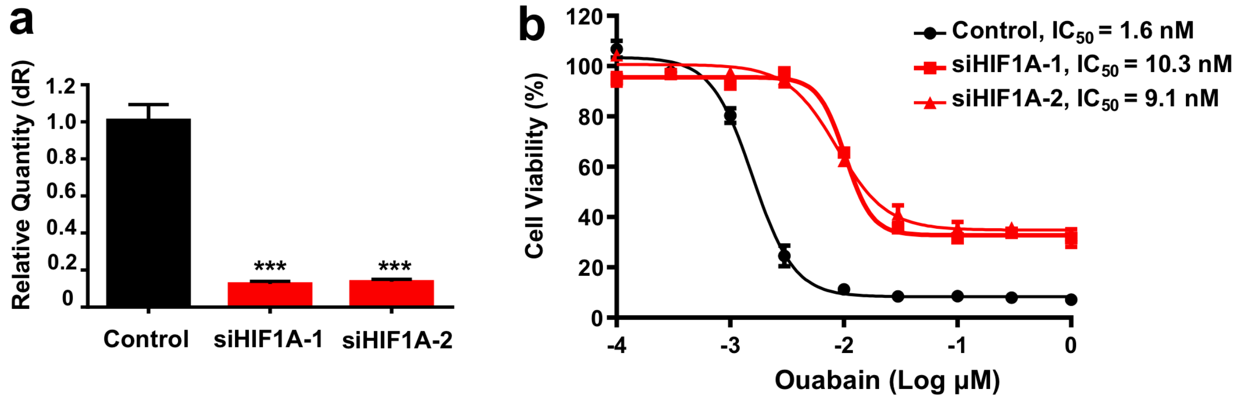

Supplementary **Fig. 13.** Ouabain's response is perturbed by HIF1A-knockdown in A549 cells. (**a**) siRNA significantly downregulates *HIF1A* gene expression in A549 cells. (**b**) Cell viability reduction by ouabain is perturbed by two specific siRNA of *HIF1A*. Each experiment was performed at least three times in duplicate and all data is represented as mean ± SEM (n = 3).

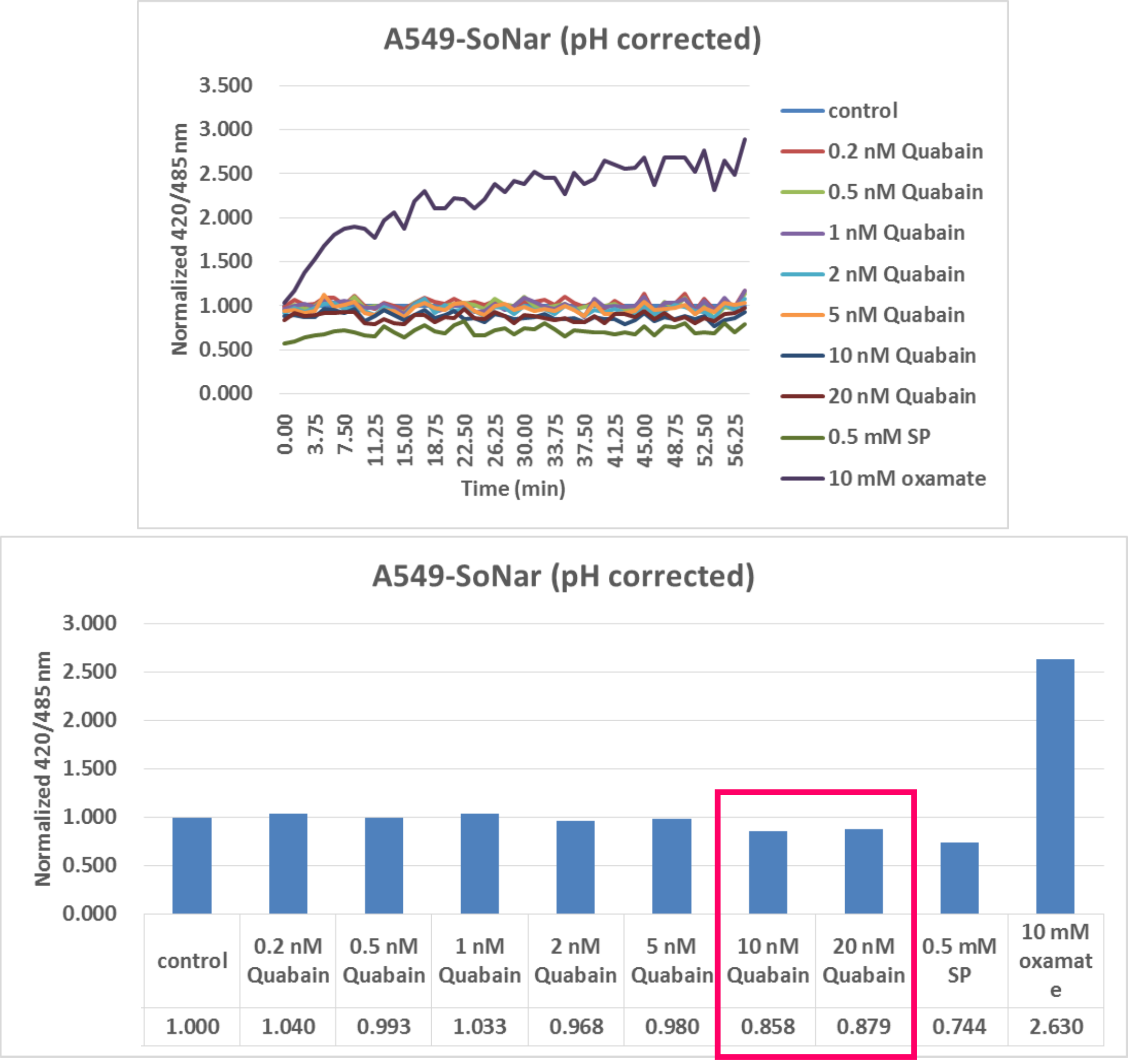

Supplementary **Fig. 14.** Effect of ouabain on NAD+/NADH ratio in A549 cell lines tested by SoNar[11]. Ouabain reduces intracellular NAD+/NADH ratio in A549 cells at 10 nM or 20 nM. Intracellular NAD+/NADH ratio was detected by SoNar fluorescence after ouabain treatment in A549 cells at 24 hour. The detailed description of this NAD+/NADH ratio assay was provided in our previous studies[11].

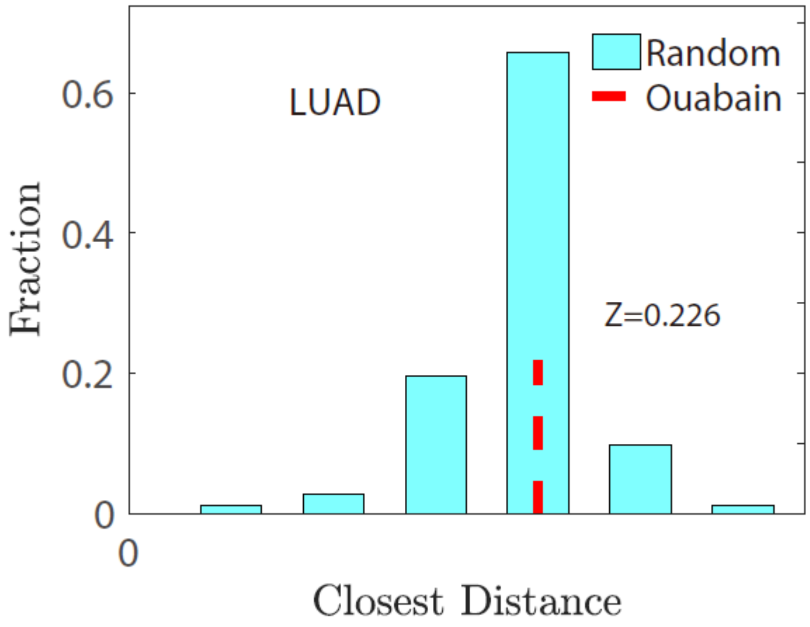

Supplementary **Fig. 15.** Network proximity analysis between ouabain's targets (red line) and the significantly mutated genes (SMGs) of LUAD comparing to the same number of randomly selected targets/genes (cyan) with a similar degree (connectivity) distribution in the human protein-protein interactome.

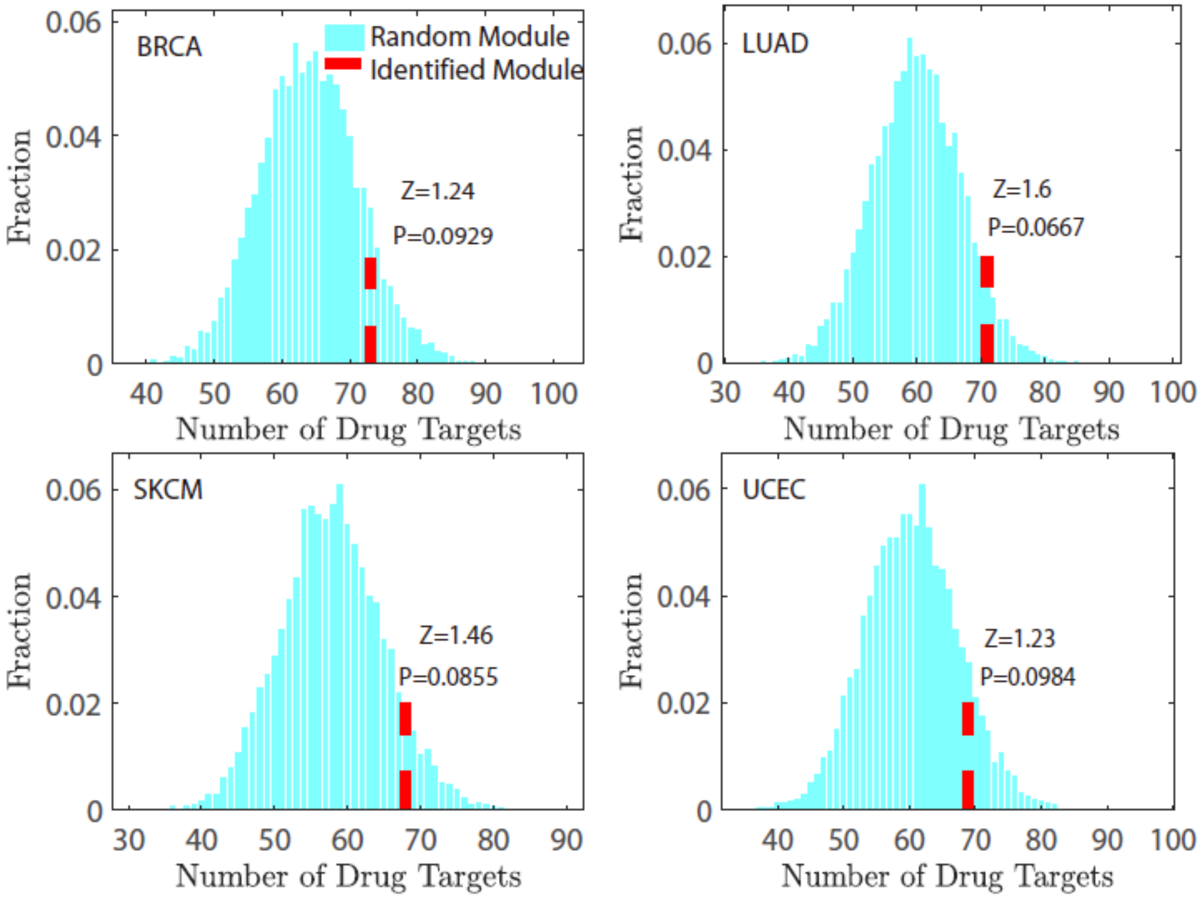

Supplementary **Fig. 16.** Enrichment analysis of drug targets in the new disease modules identified from the unbiased, systematic interactome network (https://ccsb.dana-farber.org/interactome-data.html) for four selected cancer types: invasive breast carcinoma (BRCA), lung adenocarcinoma (LUAD), skin cutaneous melanoma (SKCM), and uterine corpus endometrial carcinoma (UCEC). Z (z-score) and p-value (P) were computed by permutation test.

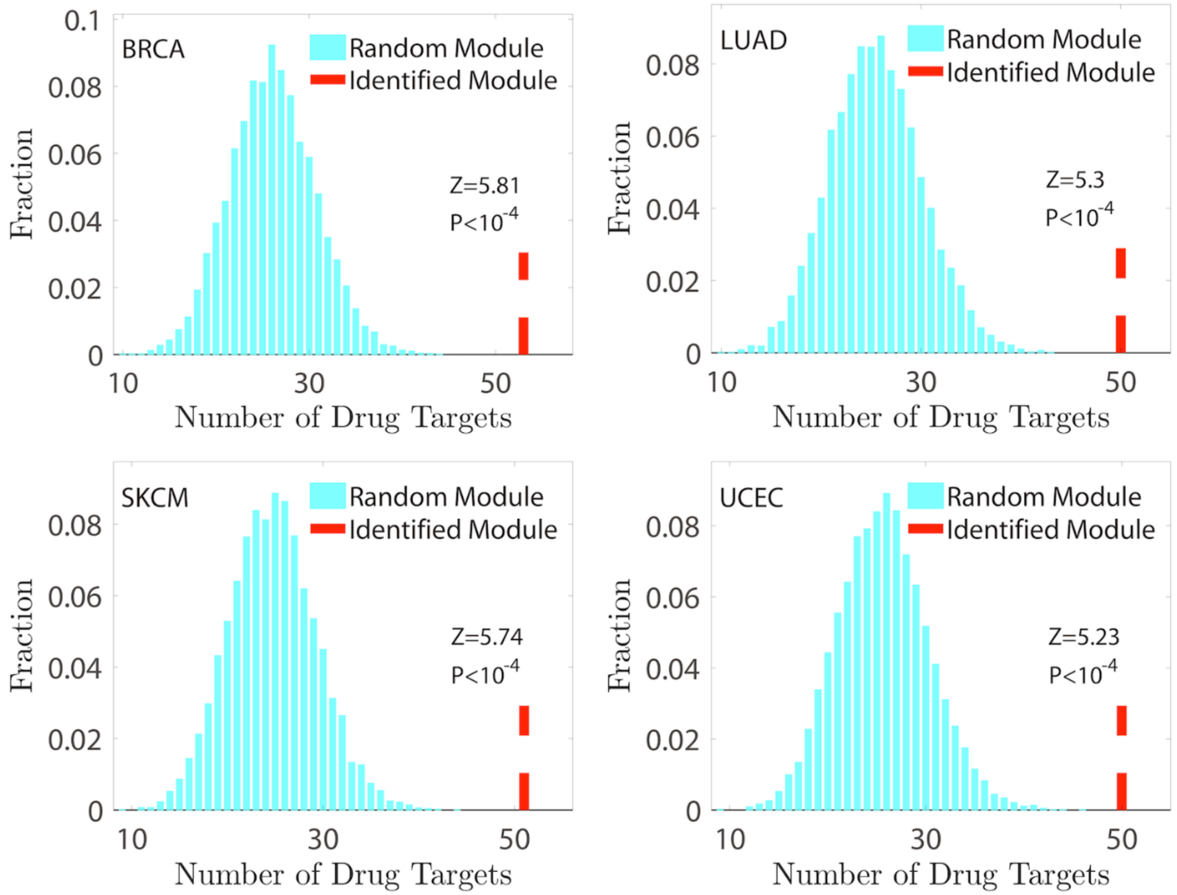

Supplementary **Fig. 17.** The enrichment analysis of drug targets in the new disease modules identified from a recently published comprehensive human binary interactome network[12] (http://interactomeinsider.yulab.org/) for four selected cancer types: invasive breast carcinoma (BRCA), lung adenocarcinoma (LUAD), skin cutaneous melanoma (SKCM), and uterine corpus endometrial carcinoma (UCEC). Z (z-score) and p-value (P) were computed by permutation test.

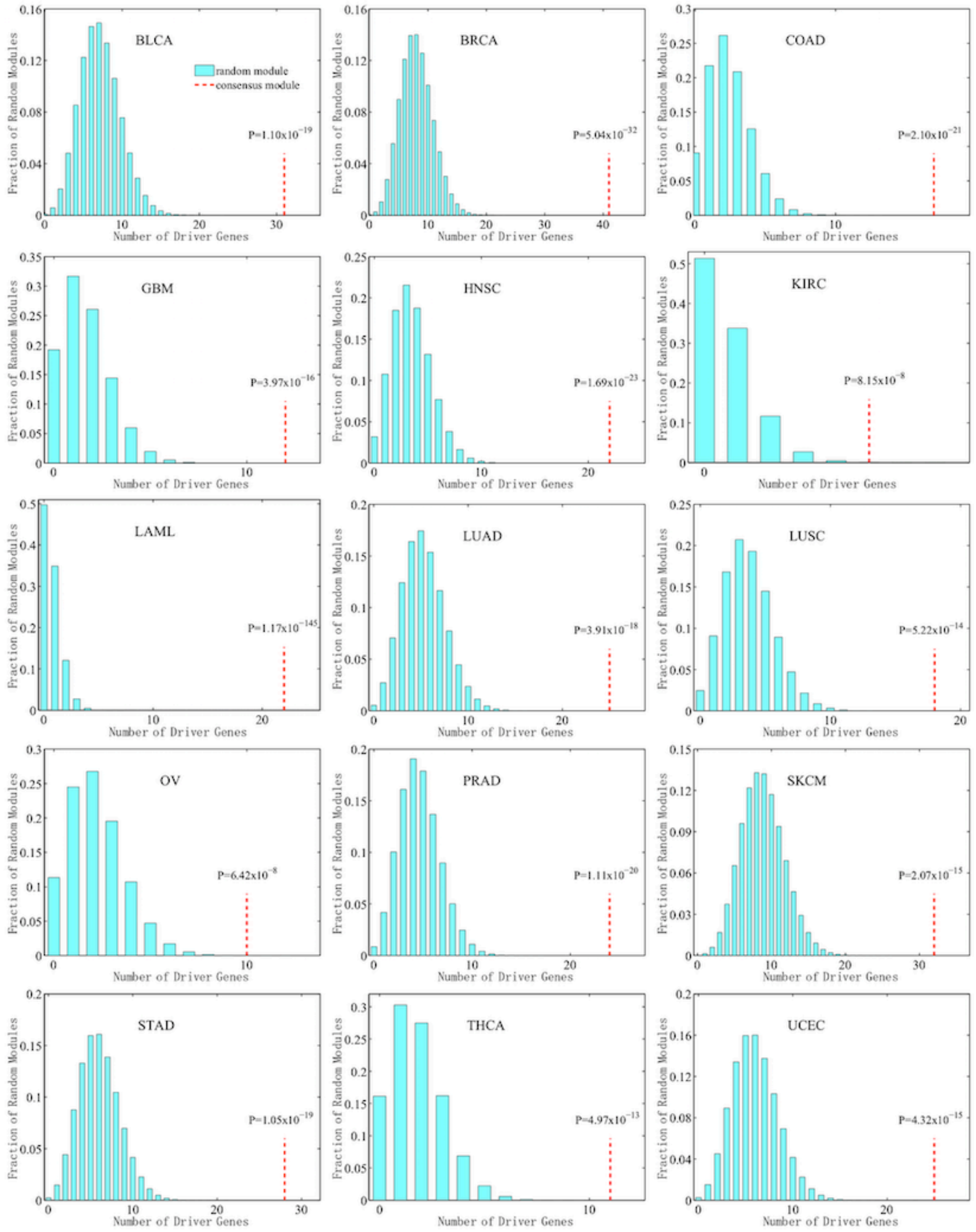

Supplementary **Fig. 18.** Known cancer driver genes (named significantly mutated genes, Supplementary **Data 1**) are appreciably enriched in patient-specific disease modules across 15 cancer types. Disease modules were identified by GPSnet when α = 0.4 (Eq. 4 and 5).

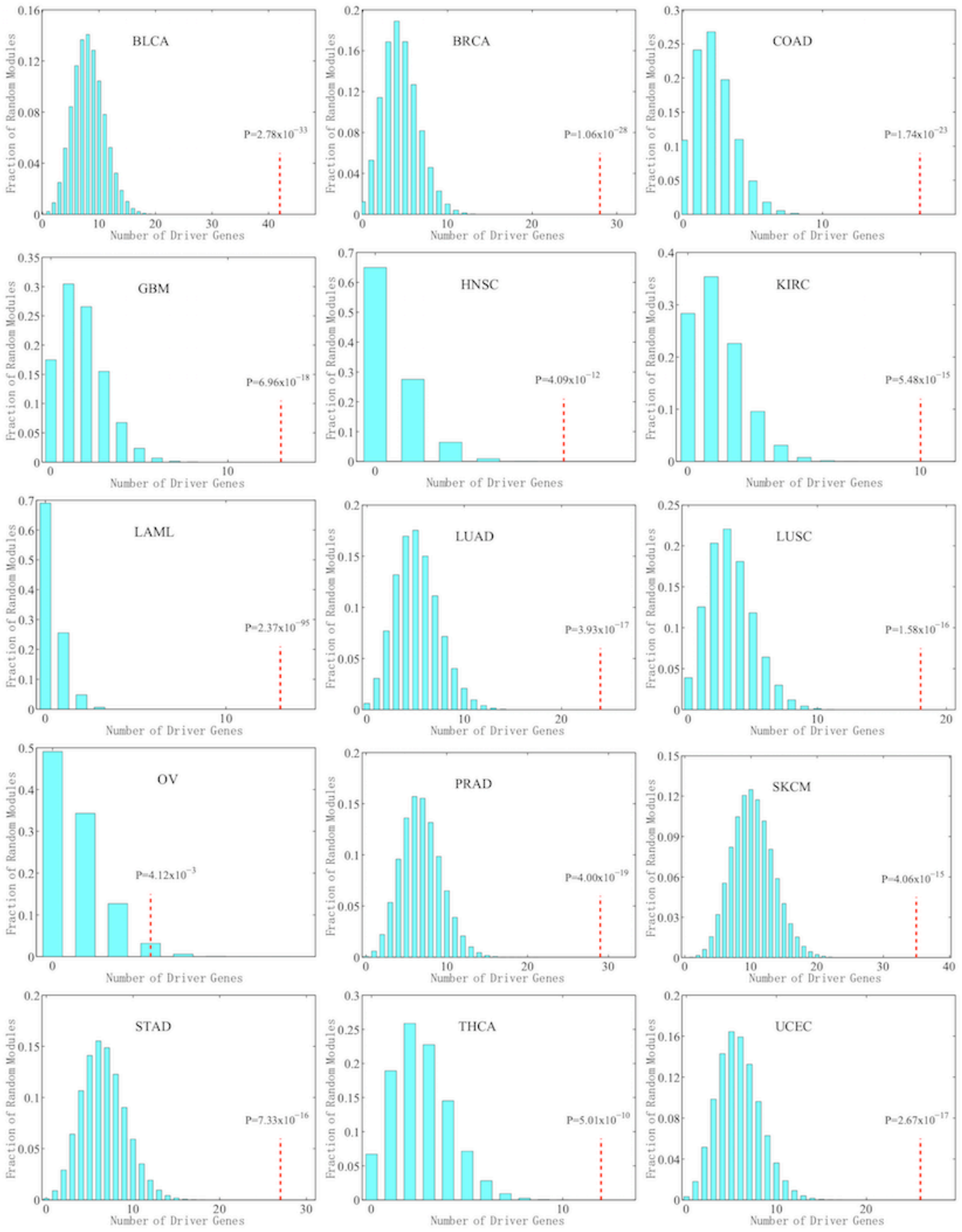

Supplementary **Fig. 19.** Known cancer driver genes (named significantly mutated genes, Supplementary **Data 1**) are appreciably enriched in patient-specific disease modules across 15 cancer types. Disease modules were identified by GPSnet when α = 0.6 (Eq. 4 and 5).

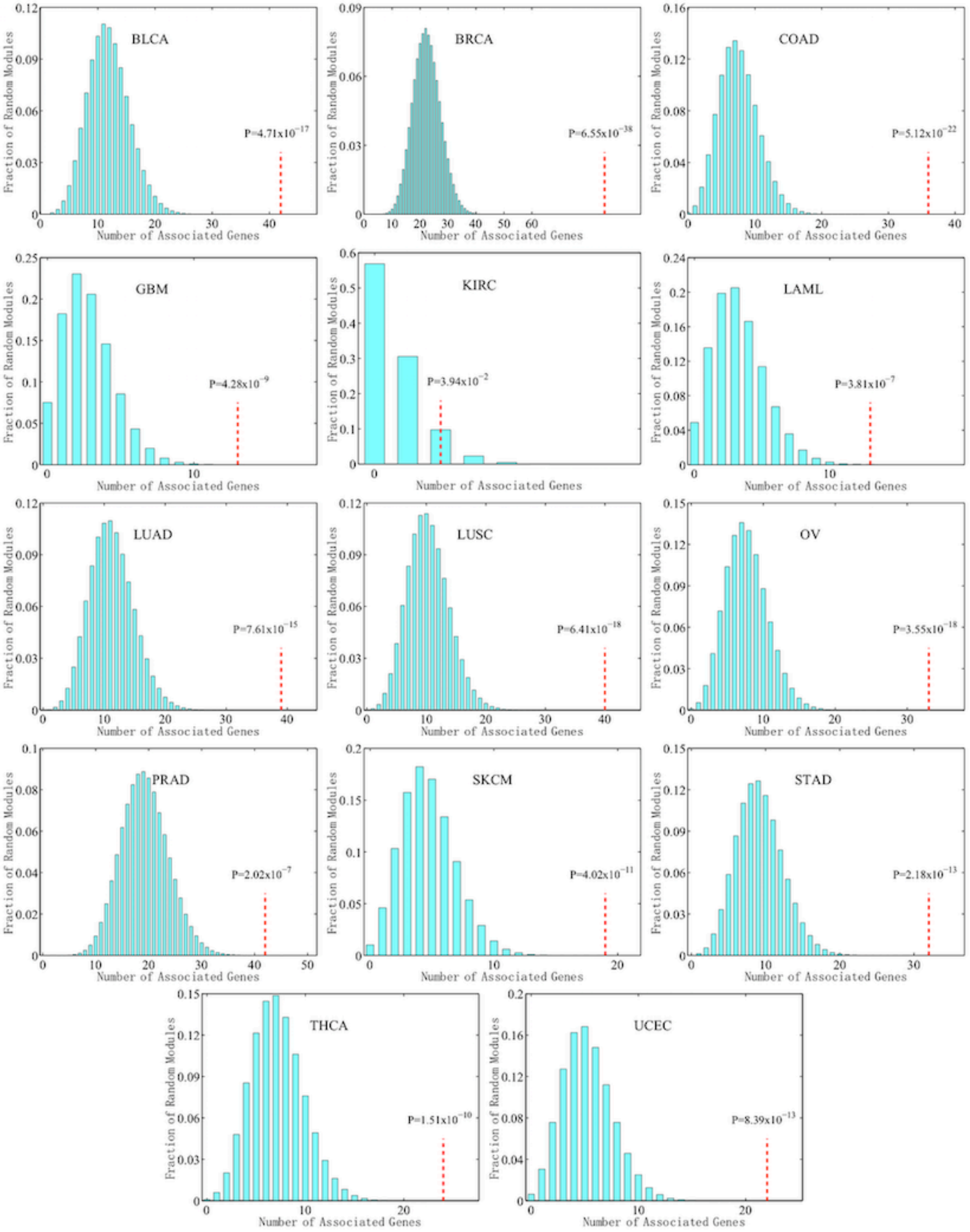

Supplementary **Fig. 20.** Known cancer-associated genes (Supplementary **Data 3**) are appreciably enriched in patient-specific disease modules across 14 cancer types. Disease modules were identified by GPSnet when α = 0.4 (Eq. 4 and 5). HNSC was excluded for validation owing to lack of known cancer-associated genes from publicly available databases.

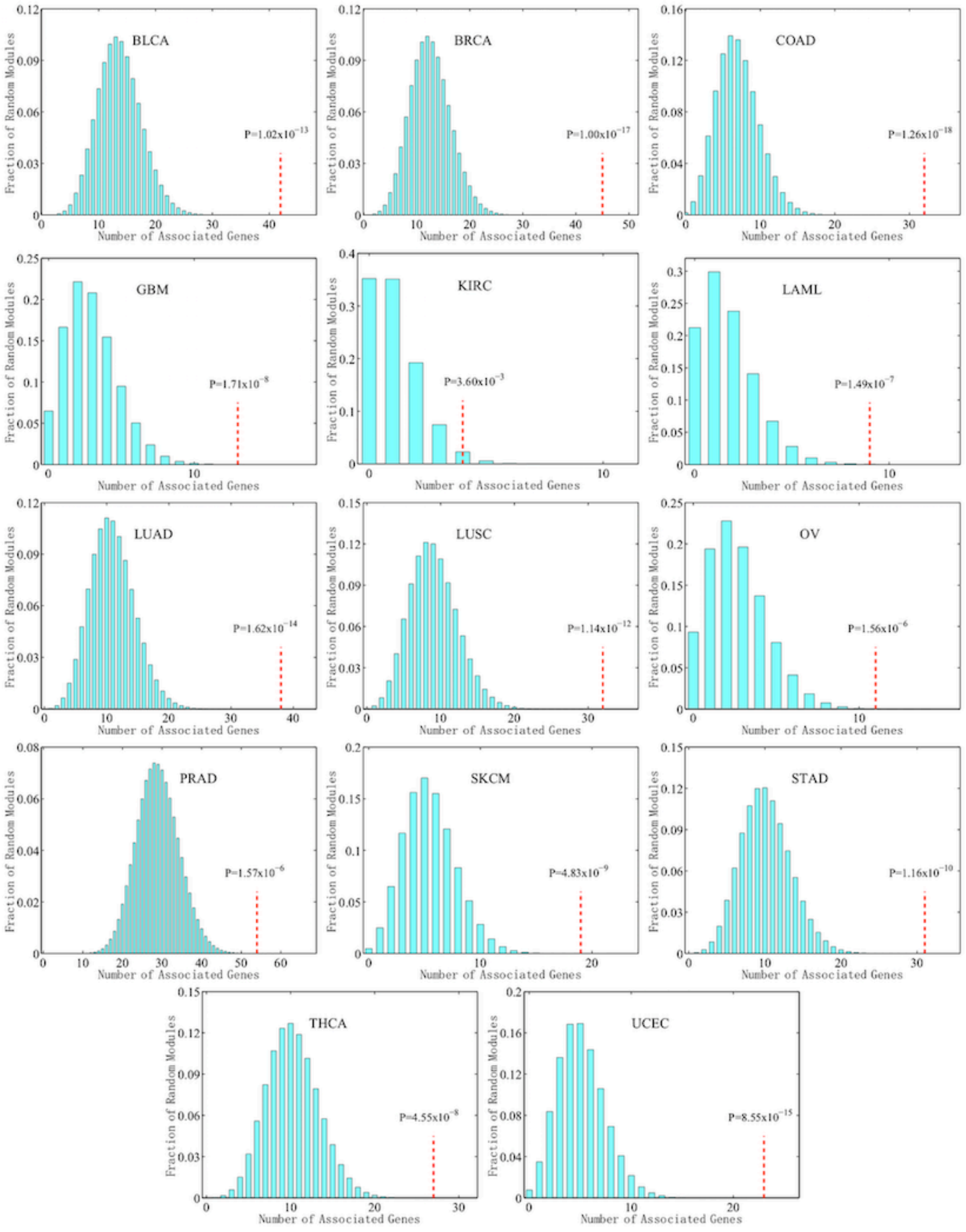

Supplementary **Fig. 21.** Known cancer-associated genes (Supplementary **Data 3**) are appreciably enriched in patient-specific disease modules across 14 cancer types. Disease modules were identified by GPSnet when $\alpha = 0.6$ (Eq. 4 and 5). HNSC was excluded for validation owing to lack of known cancer-associated genes from publicly available databases.

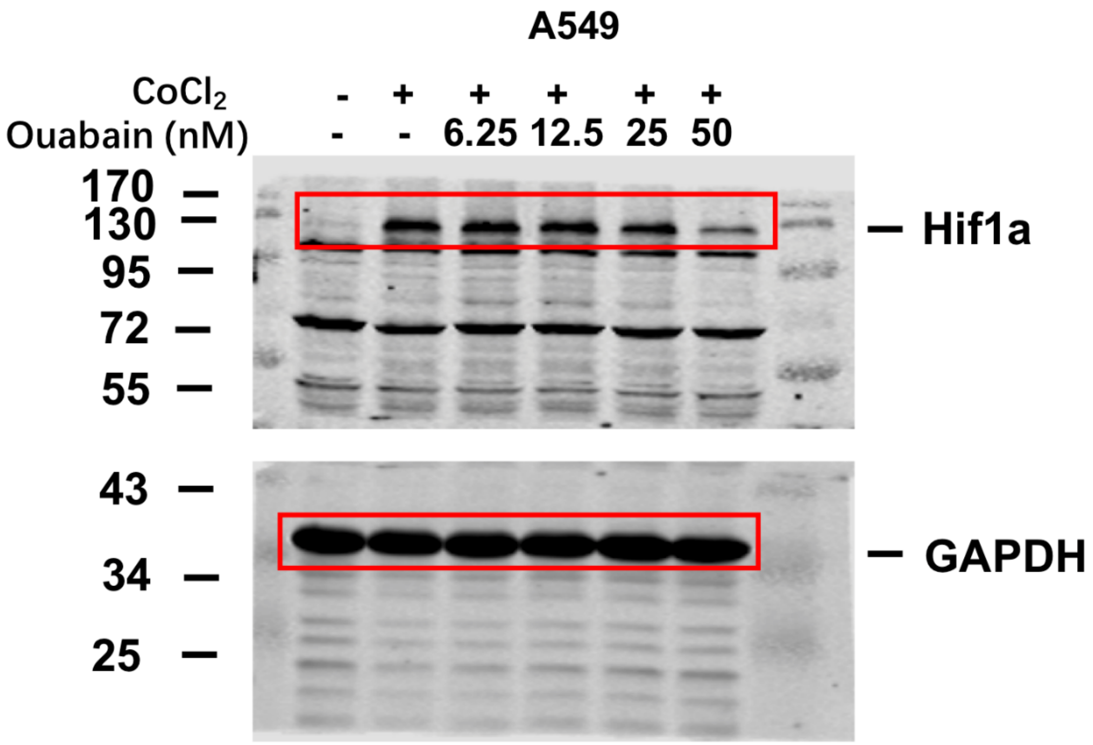

Supplementary **Fig. 22.** Uncropped images for Figure 6c Western blots. Western blot analysis of HIF1α protein expression (normalized by GAPHD) after $CoCl_2$/ouabain treatment in A549 cells (see Methods).

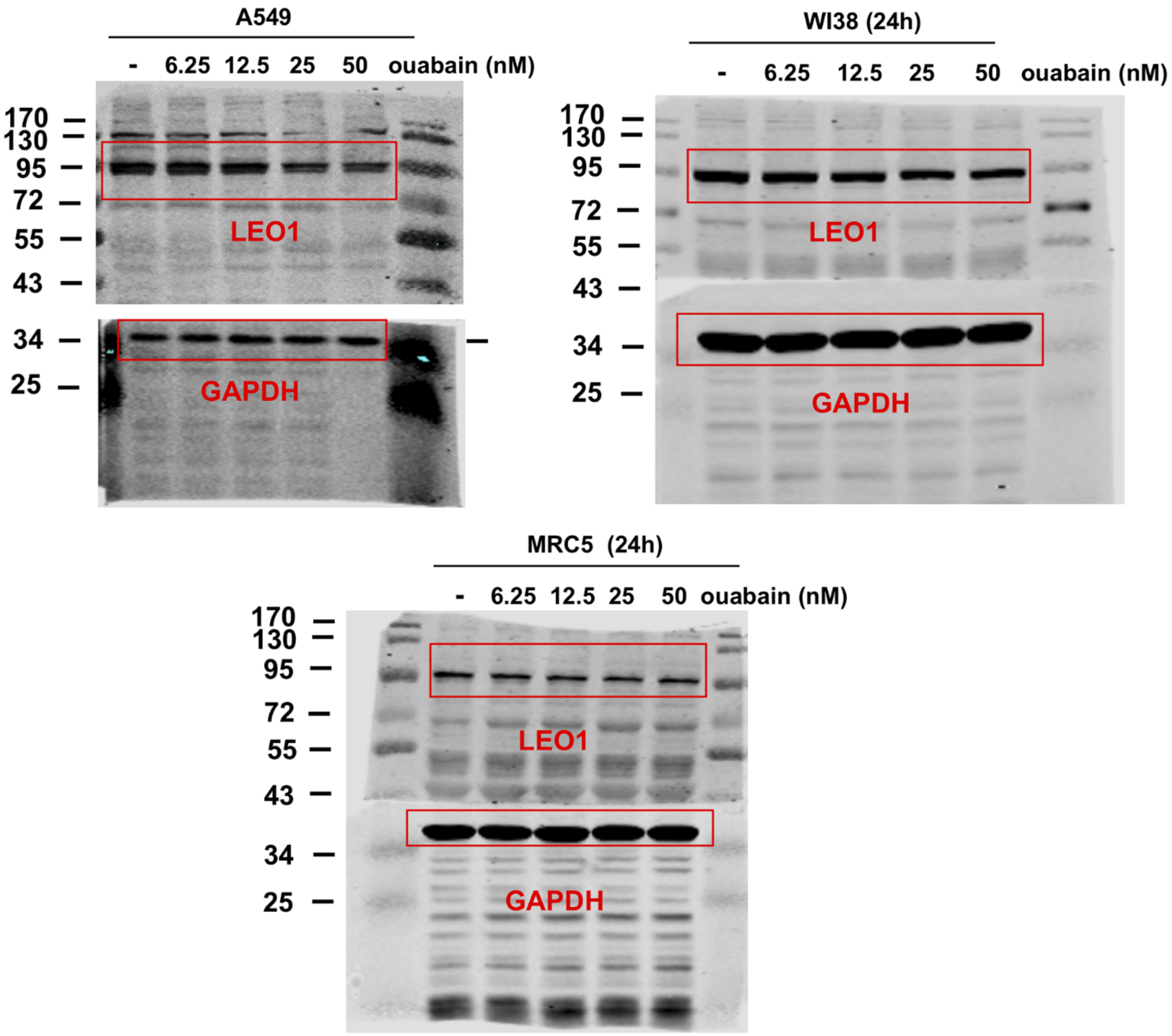

Supplementary **Fig. 23.** Uncropped images for Figure 7d Western blots. Western blot analysis of LEO1 protein expression (normalized by GAPDH) following ouabain treatment in A549 cells and two normal human lung fibroblasts cells (WI38 and MRC5).

Supplementary **Table 1**: Chemicals and reagents used in this study.

chemicals and reagents	Catalog#	Source
CellTiter 96 AQueous One Solution Cell Proliferation Assay (MTS)	G3580	Promega
DMSO (dimethylsulfoxide)	D2650	Sigma
DMEM Medium	10566016	Gibco
RPMI 1640 Medium	21875109	Gibco
MEM Medium	11095072	Gibco
FBS (Fetal Bovine Serum)	10099141	Gibco
Penicillin-Streptomycin	Vetec-V900929	Sigma-Aldrich
BSA (bovine serum albumin)	A1933	Sigma
Pierce™ BCA Protein Assay Kit	23227	Thermo-Fisher
SYBR Green Real-Time PCR Master Mixes	4334973	Thermo-Fisher
ReverTra Ace qPCR RT Master Mix	FSQ-201	TOYOBO
ExFect 2000 Transfection Reagent	T202-02	Vazyme
TRIzol™ Reagent	15596018	Invitrogen
Triton X-100	T8787	Sigma
Phosphatase Inhibitor Cocktail A	sc-45044	Santa Cruz
Phosphatase Inhibitor Cocktail B	sc-45045	Santa Cruz
Phosphatase Inhibitor Cocktail C	sc-45065	Santa Cruz
Protease Inhibitors Set	11206893001	Roche
Goat Anti-Rabbit IRDye 800CW	926-32211	LI-COR

Supplementary **Table 2**: Antibodies used in this study.

Antibodies	Catalog#	Source
anti-GAPDH	AB0037	Abways
anti-HIF-1α	CY5197	Abways
anti-LEO1	ab75721	Abcam

Supplementary **Table 3**: Primers used in this study for various PCR assays.

Genes	Forward primer (5’-3’)	Reverse primer (5’-3’)
HIF1A	ACTCAGGACACAGATTTAGACTTG	TGGCATTAGCAGTAGGTTCTTG
LEO1	AGAAGCGGATAGTGACACTGAGGT	TTCATCAACAGGCTGTCCTGGAGT
shHIF1A-1	CCGGGTGATGAAAGAATTACCGAATCTCGAGATTCGGTAATTCTTTCATCACTTTTT	
shHIF1A-2	CCGGTGCTCTTTGTGGTTGGATCTACTCGAGTAGATCCAACCACAAAGAGCATTTTT	
GAPDH	TGGCCTTCCGTGTTCCTAC	GAGTTGCTGTTGAAGTCGCA

Supplementary References

1. Menche, J. et al. Uncovering disease-disease relationships through the incomplete interactome. *Science* **347**, 1257601 (2015).
2. Rolland, T. et al. A proteome-scale map of the human interactome network. *Cell* **159**, 1212-1226 (2014).
3. Zhao, J., Cheng, F. & Zhao, Z. Tissue-specific signaling networks rewired by major somatic mutations in human cancer revealed by proteome-wide discovery. *Cancer Res.* **77**, 2810-2821 (2017).
4. Cheng, F. et al. Studying tumorigenesis through network evolution and somatic mutational perturbations in the cancer interactome. *Mol. Biol. Evol.* **31**, 2156-2169 (2014).
5. Hamosh, A., Scott, A. F., Amberger, J. S., Bocchini, C. A. & McKusick, V. A. Online Mendelian Inheritance in Man (OMIM), a knowledgebase of human genes and genetic disorders. *Nucleic Acids Res.* **33**, D514-517 (2005).
6. Yu, W., Gwinn, M., Clyne, M., Yesupriya, A. & Khoury, M. J. A navigator for human genome epidemiology. *Nat. Genet.* **40**, 124-125 (2008).
7. Hernandez-Boussard, T. et al. The pharmacogenetics and pharmacogenomics knowledge base: accentuating the knowledge. *Nucleic Acids Res.* **36**, D913-918 (2008).
8. Davis, A. P. et al. The Comparative Toxicogenomics Database: update 2011. *Nucleic Acids Res.* **39**, D1067-1072 (2011).
9. Cheng, F. et al. Network-based approach to prediction and population-based validation of *in silico* drug repurposing. *Nat. Commun.* **9**, 2691 (2018).

10. Ghiassian, S. D., Menche, J. & Barabasi, A. L. A DIseAse MOdule Detection (DIAMOnD) algorithm derived from a systematic analysis of connectivity patterns of disease proteins in the human interactome. *PLoS Comput. Biol.* **11**, e1004120 (2015).

11. Zhao, Y. et al. SoNar, a Highly Responsive NAD+/NADH Sensor, Allows High-Throughput Metabolic Screening of Anti-tumor Agents. *Cell Metab.* **21**, 777-789 (2015).

12. Meyer, M. J. et al. Interactome INSIDER: a structural interactome browser for genomic studies. *Nat. Methods* **15**, 107-114 (2018).
