# Supplementary material for: A genome-wide positioning systems network algorithm for in silico drug repurposing
Source: Nat Commun. 2019 Aug 2;10:3476. doi: 10.1038/s41467-019-10744-6 (PMC6677722; doi:10.1038/s41467-019-10744-6)
Supplement: Supplementary file 2 — Description of Additional Supplementary Files [file 41467_2019_10744_MOESM2_ESM.pdf]

Description of Additional Supplementary Files

File Name: Supplementary Data 1
Description: Known cancer driver genes, with significantly mutated genes identified that were collected from TCGA projects. (Excel file)

File Name: Supplementary Data 2
Description: Final patient-specific disease modules across 15 cancer types are identified by the GPSnet algorithm. (Excel file)

File Name: Supplementary Data 3
Description: Known cancer-associated genes were collected from four public databases: the Online Mendelian Inheritance in Man (OMIM) database, HuGE Navigator, PharmGKB, and Comparative Toxicogenomics Database (CTD). (Excel file)

File Name: Supplementary Data 4
Description: FDA-approved cancer type/subtype specific drugs. (Excel file)

File Name: Supplementary Data 5
Description: Computationally predicted anticancer indications across 15 cancer types by both network proximity and gene-set enrichment analysis approaches. (Excel file)

File Name: Supplementary Data 6
Description: Differentially expressed analysis in LUAD patients and correlation analysis between gene expression and metabolite abundance in ~70 NSCLC cell lines. (Excel file)
